# A Single *Argonaute* Gene Participates in Exogenous and Endogenous RNAi and Controls Cellular Functions in the Basal Fungus *Mucor circinelloides*

**DOI:** 10.1371/journal.pone.0069283

**Published:** 2013-07-23

**Authors:** María Cervantes, Ana Vila, Francisco E. Nicolás, Simon Moxon, Juan P. de Haro, Tamas Dalmay, Santiago Torres-Martínez, Rosa M Ruiz-Vázquez

**Affiliations:** 1 Department of Genetics and Microbiology, Faculty of Biology, University of Murcia, Murcia, Spain; 2 Regional Campus of International Excellence “Campus Mare Nostrum”, Murcia, Spain; 3 School of Biological Sciences, University of East Anglia, Norwich, United Kingdom; Universidade de Sao Paulo, Brazil

## Abstract

The mechanism of RNAi is well described in metazoans where it plays a role in diverse cellular functions. However, although different classes of endogenous small RNAs (esRNAs) have been identified in fungi, their biological roles are poorly described due, in part, to the lack of phenotype of mutants affected in the biogenesis of these esRNAs. Argonaute proteins are one of the key components of the RNAi pathways, in which different members of this protein family participate in the biogenesis of a wide repertoire of esRNAs molecules. Here we identified three *argonaute* genes of the fungus *Mucor circinelloides* and investigated their participation in exogenous and endogenous RNAi. We found that only one of the *ago* genes, *ago-1,* is involved in RNAi during vegetative growth and is required for both transgene-induced RNA silencing and the accumulation of distinct classes of esRNAs derived from exons (ex-siRNAs). Classes I and II ex-siRNAs bind to Ago-1 to control mRNA accumulation of the target protein coding genes. Class III ex-siRNAs do not specifically bind to Ago-1, but requires this protein for their production, revealing the complexity of the biogenesis pathways of ex-siRNAs. We also show that *ago-1* is involved in the response to environmental signals, since vegetative development and autolysis induced by nutritional stress are affected in *ago-1* ^−^ *M. circinelloides* mutants. Our results demonstrate that a single Ago protein participates in the production of different classes of esRNAs that are generated through different pathways. They also highlight the role of ex-siRNAs in the regulation of endogenous genes in fungi and expand the range of biological functions modulated by RNAi.

## Introduction

RNA silencing or RNA interference (RNAi) has been largely known as a defence mechanism against invasive nucleic acids, such as viruses, transposons or transgenes, in a wide spectrum of eukaryotic organisms [1]. It acts through short interfering RNA molecules (siRNAs) that are generated from double stranded RNA (dsRNA) precursors derived from the exogenous sequences by the RNaseIII Dicer. These siRNAs are bound to an Argonaute protein within the RNA-induced silencing complex (RISC), where they serve as a guide to identify complementary target RNA molecules for silencing or destruction [2], [3]. In metazoans, RNAi also has a role in the regulation of endogenous functions through several classes of endogenous small RNA (esRNAs) molecules, which are generated from genome-encoded precursors. These endogenous pathways play many fundamental roles, including regulation of mRNA accumulation and translation, chromatin silencing, programmed DNA rearrangements and genome surveillance [3]. In fungi, recent reports have described the existence of different esRNA classes that are generated by different RNAi pathways [4], although the lack of clear phenotypes in the majority of mutants affected in genes required for the esRNA biogenesis has hampered the identification of the physiological roles of the fungal RNA silencing pathways.

*Mucor circinelloides* is an emerging opportunistic human pathogen that has recently attracted the interest of the scientific community due to the increase of the lethal fungal infection mucormycosis, which preferentially affects immunocompromised patients and whose causal agents are *M. circinelloides* and other basal zygomycete fungi [5], which are the ancient class of terrestrial saprophytic fungi. The availability of molecular tools and its evolutionary distance from other fungal model organisms, such as *Neurospora crassa*, have made *M. circinelloides* a model organism in the fungal kingdom for the study of different molecular processes, including fungal pathogenesis [6], RNA silencing and other processes. Transgene-induced RNA silencing in *M. circinelloides* is associated with the accumulation of two size classes of siRNAs, 21 nt and 25 nt long, which are differentially accumulated during the vegetative growth [7]. Only one of the two *dicer* genes that have been identified in *M. circinelloides*, *dcl-2*, is required for transgene-induced silencing and accumulation of the two size classes of siRNAs, both when silencing is induced by sense and inverted repeat transgenes [8], [9]. Unlike *N. crassa*, RNA silencing in *M. circinelloides* is associated with an amplification step that generates secondary siRNAs corresponding to target sequences by the RNA-dependent RNA polymerase activity of the *rdrp-2* gene product [10]. A functionally distinct *rdrp* gene, *rdrp-1*, is essential for initiation of silencing by sense transgenes by producing antisense RNA transcripts derived from the transgene, but it is not involved in the amplification of the silencing signal [10].

The *M. circinelloides* RNAi pathway also operates to produce esRNAs [11]. Deep sequencing of sRNAs endogenously accumulated in the wild type strain and *dicer* ^−^ and *rdrp* ^−^ mutants identified new classes of esRNA that map to exons and regulate the expression of many protein coding genes. These esRNAs were named exonic-siRNAs (ex-siRNAs) and were classified in four different classes based on the silencing proteins required for their biogenesis. Classes I and II include all ex-siRNAs that are *dcl-2*-dependent. Class II, which is the largest one, includes 222 exons and also requires the *rdrp-1* gene product, whereas class I, which includes only nine exons, is *rdrp-1*-independent [11]. Class III corresponds to ex-siRNAs that can be generated by either Dcl-1 or Dcl-2 and require *rdrp-1* and *rdrp-2* gene products, and the class IV is a tiny group of ex-siRNAs that depend on *dcl-1* but not *dcl-2*. The different biogenesis requirement of the ex-siRNAs highlights the complexity of the endogenous RNA silencing pathways in fungi.

Both the exogenously-induced RNAi pathway and the endogenous production of functional esRNAs in *M. circinelloides* must require the involvement of Argonaute (Ago) proteins, although no *ago* genes have been identified to date in this fungus. The Argonaute protein family was first identified in plants, and members are defined by the presence of PAZ (Piwi-Argonaute-Zwille), MID (Middle) and PIWI domains [12], [13]. The N-terminal PAZ domain contains a specific binding pocket that anchors the characteristic two-nucleotide 3' overhang that results from digestion of dsRNAs by Dicer [14]. The MID domain binds the characteristic 5′ phosphates of sRNAs, anchoring these molecules onto the Ago protein [15]. Finally, the PIWI domain of Ago proteins is the catalytic centre for rendering their target cleavage activity similar to RNase H. It contains the catalytic triad DDH, where the aspartate residues are invariant whereas the third residue can be substituted by other residue [12], [13]. However, the sole presence of the catalytic triad does not ensure the slicer activity, since many Argonaute proteins containing the catalytic residues are endo-nucleolytically inactive. It has been speculated that other factors, such as post-translational modifications, might contribute to the slicer activity [12].

Argonaute proteins are highly conserved between species and many organisms encode multiple members of the family. Functional analysis of Ago proteins shows that the RISC variants can be distinguished by their Ago proteins, which participate in specific RNA silencing pathways. In this work we describe the identification and functional analysis of three different *ago* genes in *M. circinelloides*. Only the *ago-1* gene is required for exogenously-induced RNA silencing or for the endogenous production of sRNAs during vegetative development. The four classes of ex-siRNAs are down-regulated in the *ago-1* ^−^ mutant strain relative to the wild type, although only Classes I and II are specifically bound to Ago-1, suggesting that they are *bona fide* siRNAs that target mRNAs of specific genes. Phenotypic analysis of the *ago-1* ^−^ mutant indicates that *ago-1* is involved in the regulation of asexual development and in the lytic response to nutritional starvation, pointing out the physiological role of the RNA silencing mechanism in this basal fungus.

## Results

### Three *argonaute* Genes in the *M. circinelloides* Genome

**Figure 1:**
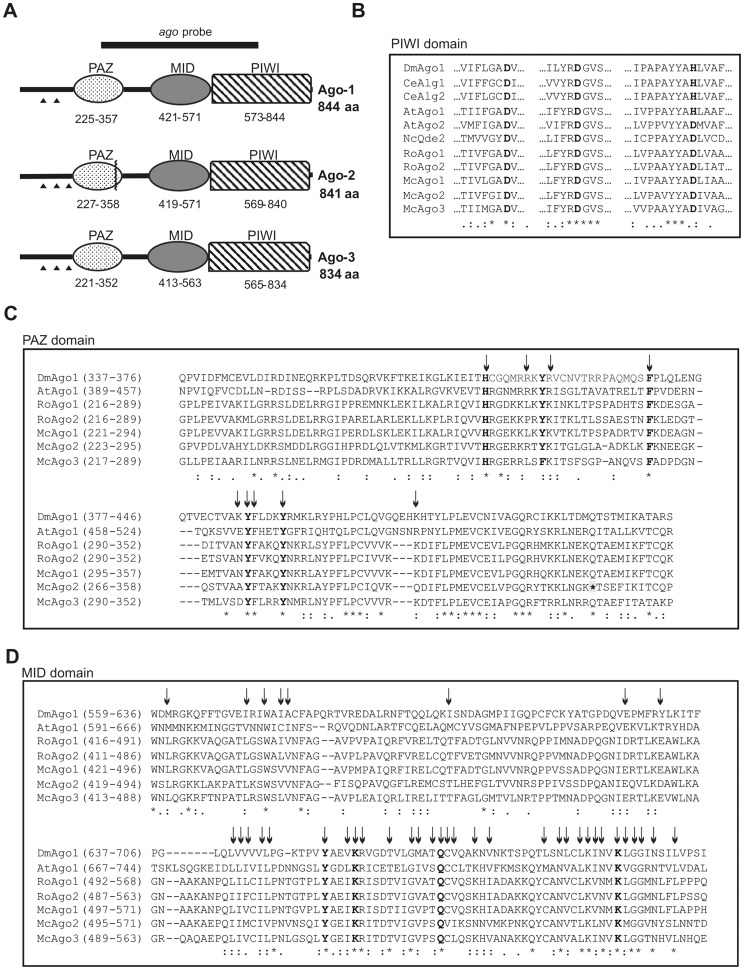
Domain structure of the *M. circinelloides* Ago proteins. **A.** Domain organization of the Ago-1, Ago-2 and Ago-3 proteins. Domains are shown by boxes with the starting and stopping amino acid of each domain indicated. PAZ, Piwi/Argonaute/Zwille domain; MID, Middle domain; PIWI, RNase H-type PIWI domain. Positions of introns in the *ago* genes are indicated by arrowheads. Position of the stop codon in Ago-2 is indicated by a zigzag line. The probe used in the screening of the genomic library to clone the *ago* genes is shown above the scheme (*ago* probe). **B.** Amino acid sequence alignment of parts of PIWI domains encompassing the three catalytic residues. The DDD/E/H/K motif is shown in bold. **C.** Amino acid sequence alignment of the PAZ domain of *M. circinelloides* Ago proteins and several Ago proteins from different organisms. * indicates the stop codon of *M. circinelloides* Ago-2. Arrows indicate the amino acids whose mutations led to a decreased RNA-binding efficiency [14]. In bold, residues that have been proposed to form the binding pocket for the 2-nucleotide 3′-overhang of the siRNA duplex [13], [14]. **D.** Amino acid sequence alignment of the MID domain of *M. circinelloides* Ago proteins and several Ago proteins from different organisms. Arrows indicate conservative residues among 29 Ago proteins previously analyzed [13]. Essential residues for binding the 5′ terminal nucleotide of the guide sRNAs molecules are shown in bold. In B, C and D, identical residues are indicated by asterisks and similar residues by points and colons. The sequences used in the alignments were *Drosophila melanogaster* (Dm) Ago1 (NP_725341); *Arabidopsis thaliana* (At) Ago1 (NP_849784) and Ago2 (NP_174413); *Caenorhabditis elegans* (Ce) Alg1 (NP_510322) and Alg2 (NP_871992); *Neurospora crassa* (Nc) Qde2 (AAF43641.1); *Rhizopus oryzae* (Ro) Ago1 (RO3G_13047.3) and Ago2 (RO3G_10137.3). Accession numbers are for GenBank except for *R. oryzae* (Broad Institute, Cambridge, MA).

Three different *argonaute*-like (*ago*) genes of *M. circinelloides*, *ago-1*, *ago-2* and *ago-3*, were cloned by screening a genomic Lambda-GEM-11 library of the wild-type strain of *M. circinelloides* with a probe corresponding to a 966 bp fragment of the *ago-1* gene amplified with degenerated oligonucleotides (Fig. 1A, probe a) (see Materials and Methods). Comparison between the cDNAs and the genomic sequences of *ago-1* and *ago-2* identified two and three introns in these genes, respectively (Fig. 1A). Sequence analyses predicted the existence of three conserved introns in *ago-3*, although experimental confirmation was not possible, probably due to its extremely low level of expression during vegetative growth (see below).

The protein sequences deduced from the three *ago* genes showed a high degree of similarity among them and with other Ago proteins of zygomycetes (Fig. S1A). The *M. circinelloides* Ago-1 protein shows higher similarity to the zygomycete proteins than to its paralogous Ago-2 and Ago-3 proteins, suggesting that they derive from different ancestors. This is apparent when the phylogenetic relationships among Argonaute proteins of zygomycetes are analyzed (Fig. S1B). The *M. circinelloides* Ago proteins include all the structural domains of the Argonaute protein family: a siRNA-binding PAZ domain, a middle domain and an RNase H-type PIWI domain (Fig. 1A). The catalytic residues DDH/D/E/K of the PIWI domain has been identified in the three *M. circinelloides* protein sequences, with all the fungal proteins containing the triad DDD (Fig. 1B). They also contain all the PAZ residues proposed to form the binding pocket that anchors the characteristic two-nucleotide 3′ overhang of the siRNA that results from digestion of dsRNAs by Dicer (Fig. 1C). Additionally, the *M. circinelloides* Ago proteins contain all the essential residues of the functionally important MID domain, which binds the 5′ phosphates of siRNAs and thus anchors siRNAs onto the Ago protein (Fig. 1D). The presence of all the conserved residues in the Ago-2 protein is especially remarkable, since genomic and cDNA analyses of the *ago-2* gene demonstrated the presence of an in frame stop codon within the sequence that would produce a truncated protein lacking the MID and PIWI domains (Fig. 1A). This mutation is fixed in the parental wild-type strain CBS 277.49, which suggests that *ago-2* is a pseudogene, although the sequence conservation indicates that the mutation event has occurred recently.

### Expression Analysis of the *argonaute* Genes

**Figure 2:**
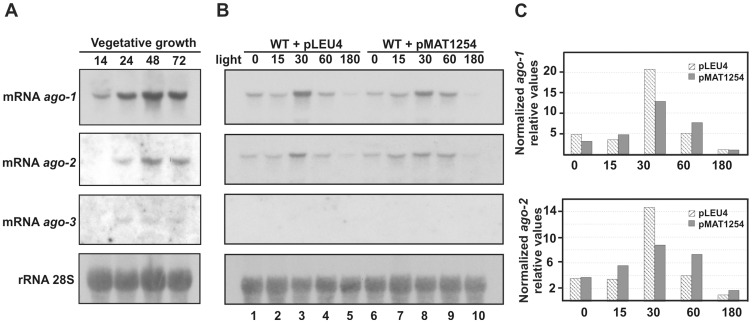
Expression of the *ago* genes in *M. circinelloides*. **A.** Northern blot analysis of total RNA isolated from the wild type strain R7B grown for different time (hours) in liquid minimal medium. The filter was hybridized with specific probes for each of the *ago* genes (see Fig. S2), and reprobed with a 28S rRNA probe to check loading. **B.** Northern blot analysis of total RNA isolated from the wild-type strain containing the control plasmid pLEU4 (lanes 1 to 5) or the dsRNA-producing vector pMAT1254 (lanes 6 to 10). Cultures were grown in liquid medium under dark conditions during 24 h (time 0) and then illuminated with continuous light for different periods of time (minutes). The filter was hybridized with the same probes as in A, checking for the lack of signals before each hybridization. Similar results were obtained in two different experiments. **C.** Densitometric analysis of expression data shown in B. Signal intensities were quantified and normalized to rRNA levels. All data were again normalized with respect to the expression value of the control strain (pLEU4) after 180 min of illumination.

The expression profiles of the *ago* genes during vegetative growth were analyzed by Northern blot experiments. Total RNA was isolated from the wild type strain and hybridized under stringent conditions with *ago*-specific probes that were demonstrated to show no cross-hybridization among the three *ago* genes (Fig. S2; see Materials and Methods). Results indicated that the three genes were expressed during the vegetative growth of the wild-type strain, although at different levels (Fig. 2A). The amount of *ago-1* and *ago-2* transcripts increased similarly during the vegetative cycle, with maximum accumulation after 48 h of growth, when the liquid cultures reached the end of the exponential phase, showing that the putative pseudogene *ago-2* displayed a transcription pattern similar to that of the *ago-1* gene. In contrast, accumulation of the *ago-3* mRNA during the vegetative cycle was barely observed, being only detected after overexposure of the membranes. We also investigated if the expression of these genes was induced by the presence of dsRNA molecules. For that, a hairpin RNA construct expressed by the regulated *carB* promoter, which is inducible by light (pMAT1254; [9]) was introduced into the wild-type strain and the level of *ago* transcripts was monitored after different times of incubation under light conditions. As a control, a wild type strain harbouring an empty vector was also analyzed (Fig. 2B). Unexpectedly, the *ago-1* and *ago-2* mRNA accumulation observed in the strain expressing the hairpin RNA construct (pMAT1254) under light conditions was similar to that observed in the control strain (pLEU4) (Fig. 2C), suggesting that the increase in the *ago-1* and *ago-2* mRNA accumulation is due to their activation by light and not to the presence of dsRNA molecules. The *ago-3* expression could not be detected in these assays.

### Generation of Knockout Mutants for the *argonaute* Genes

Null mutants were generated for each *ago* gene by gene replacement, designing a knockout vector to disrupt each gene (see description of plasmids in Materials and Methods S1). These vectors contained the *pyrG* gene, used as a selective marker, flanked by sequences of the corresponding *ago* genes and adjacent regions. Restriction fragments from each plasmid containing the *pyrG* gene and sufficient sequences of the *ago* genes to allow homologous recombination were used to transform the MU402 strain, which is auxotrophic for uracil and leucine. Homokaryotic transformants that correctly integrated the knockout vector were identified by PCR analysis (Materials and Methods S1). One out of 7 homokaryotic transformants obtained with the *ago-1* disruption fragment amplified the expected fragment for *ago-1* mutation and was named MU413. Two out of 5 homokaryotic transformants amplified the expected fragment for *ago-2* mutation and were named MU416 and MU417, and two out of 12 homokaryotic transformants amplified the expected fragment for *ago-3* mutation and were named MU414 and MU415. The disruption of each gene was confirmed by Southern analysis (Fig. S3 and Materials and Methods S1).

### The *ago-1* Gene is Absolutely Required for Exogenous Gene Silencing

The capacity of the *ago* mutants to activate the silencing mechanism by exogenous sequences was investigated using two different self-replicative silencing vectors containing sequences of the *carB* gene, which is required for the synthesis of coloured carotenoids. Plasmid pMAT647 carries a sense *carB* transgene (s-transgene) expressed from its own light-inducible promoter as a silencing reporter, and the *leuA^+^* gene as a selectable marker [7]. Plasmid pMAT1253 expresses a *carB* hairpin RNA (hpRNA) under the control of the *M. circinelloides gpd1* promoter [9]. Both plasmids are able to silence the endogenous *carB* expression when introduced into the wild-type strain, giving rise to a high proportion of transformants that remain albino in the light because of the lack of the *carB* function (Table 1). Most of the silenced primary transformants exhibited patches of albino and wild-type (bright yellow) phenotype, which is due to the presence of several nuclei in *M. circinelloides* protoplasts, but they turned uniformly albino after a cycle of vegetative growth in selective medium that increase the proportion of transformed nuclei. The hpRNA-expressing plasmid is a more efficient silencing trigger than the sense transgene construct, with silencing efficiencies ranging from 76 to 95% for the hpRNA construct and from 34 to 53% for the sense transgene (Table 1 and Table 2). These differences in the silencing efficiency have been previously reported and are due to the different strength of the promoters and the different structures of the constructs [9]. However, when pMAT647 and pMAT1253 were separately introduced into the *ago-1* ^-^ mutant, no albino transformants could be found, either in the initial transformants or after they were grown for a vegetative cycle in selective medium to increase the proportion of transformed nuclei (Table 1). These results indicate that *ago-1* plays an essential role in the RNA silencing mechanism triggered by sense or inverted repeat transgenes. In contrast, *ago-2* and *ago-3* genes do not seem to participate in the transgene-induced silencing mechanism during the mycelial phase of vegetative development. Transformations of two independent *ago-2^−^* and two independent *ago-3^−^* null mutants with pMAT647 and pMAT1253 gave rise to albino transformants at similar frequencies to the wild-type strain (Table 2). These results indicate that neither the transcribed pseudogene *ago-2* nor the *ago-3* gene are involved in vegetative RNA silencing triggered by exogenous transgenes, although we cannot discard a role for those genes under different developmental conditions.

**Table 1:** Gene silencing by self-replicative sense and inverted repeat transgenes in the *ago-1* ^−^ mutant MU413^a^.

		No. of transformants			
Plasmid^b^	Strain	Albino	Bright yellow	Total	Silencing frequency (%)
pMAT647 (s-transgene)	wild-type	34	66	100	34
	MU413 (*ago1* ^−^)	0	300	300	0
pMAT1253 (hpRNA)	wild-type	104	33	137	75.9
	MU413 (*ago1* ^−^)	0	358	358	0
pMAT1337 (hpRNA/*ago-1^+^*)	wild-type	189	10	199	95
	MU413 (*ago1* ^−^)	187	13	200	93.5
pLEU4 (control)^c^	wild-type	0	102	102	0
	MU413 (*ago1* ^−^)	0	126	126	0

a The colours of the *M. circinelloides* transformants were observed after 48 h under illumination with white light. Colonies with patches of albino and wild type phenotype were considered as albino.

b hpRNA, construct expressing hpRNA; s-transgene, construct expressing the sense transgene.

c Control plasmid without a silencing construct.

**Table 2:** Gene silencing by self-replicative sense and inverted repeat transgenes in the *ago-2* ^−^ and *ago-3* ^−^ mutants^a^.

		No. of transformants			
Plasmid^b^	Strain	Albino	Bright yellow	Total	Silencing frequency (%)
pMAT647 (s-transgene)	wild-type	92	108	200	46
	MU416 (*ago2* ^−^)	95	99	194	49
	MU417 (*ago2* ^−^)	122	108	230	53
	MU414 (*ago3* ^−^)	81	118	199	40.7
	MU415 (*ago3* ^−^)	74	122	196	36.8
pMAT1253 (hpRNA)	wild-type	306	38	344	89
	MU416 (*ago2* ^−^)	219	11	230	95.2
	MU417 (*ago2* ^−^)	172	17	189	91
	MU414 (*ago3* ^−^)	186	14	200	93
	MU415 (*ago3* ^−^)	190	10	200	95
pLEU4 (control)^c^	wild-type	0	127	127	0
	MU416 (*ago2* ^−^)	0	27	27	0
	MU417 (*ago2* ^−^)	0	28	28	0
	MU414 (*ago3* ^−^)	0	25	25	0
	MU415 (*ago3* ^−^)	0	28	28	0

a The colours of the *M. circinelloides* transformants were observed after 48 h under illumination with white light. Colonies with patches of albino and wild type phenotype were considered as albino.

b hpRNA, construct expressing hpRNA; s-transgene, construct expressing the sense transgene.

c Control plasmid without a silencing construct.

The silencing-defective phenotype shown by the *ago-1* ^−^ strain MU413 was exclusively due to the absence of the *ago-1* gene, as it was confirmed by complementation of the mutant phenotype by the *ago-1* wild-type allele. For that, a plasmid containing the complete *ago-1* gene and regulatory sequences and a *carB* inverted repeat transgene as silencing trigger (pMAT1337, Materials and Methods S1) was used to transform the MU413 mutant strain. This plasmid should simultaneously allow the recovering of the *ago-1* function and the induction of silencing by the inverted repeat transgene. In fact, the frequency of albino colonies among the resultant transformants was similar to the wild-type strain (Table 1), showing that the wild-type *ago-1* allele fully complements the mutant phenotype of the *ago-1^−^* strain.

### Accumulation of Transgenic siRNAs Depends on *ago-1*

**Figure 3:**
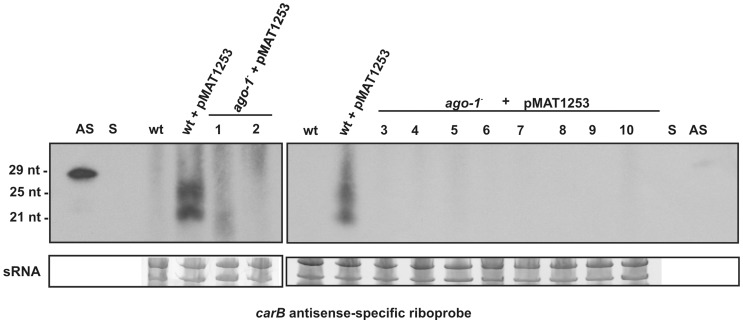
siRNA production in the *ago-1* ^−^ *M. circinelloides* mutant. Northern blot analysis of low molecular weight RNA (50 µg) isolated from wild-type silenced strains transformed with plasmid pMAT1253 (hpRNA) and 10 non-silenced *ago-1* ^−^ transformants containing the same plasmid. The RNA blot was hybridized with a *carB*-antisense specific riboprobe (pMAT652; [7]), which contains a 1662 bp fragment of the *carB* gene in the sense orientation that is able to detect transgenic and endogenous *carB* sequences. The cultures were grown for 24 h in liquid medium under continuous illumination conditions. One picomol per lane of 29-mer DNA oligonucleotides in antisense orientation (AS) and 25-mer oligonucleotide in sense orientation (S) were used as controls. The predominant RNA species in the small-RNA samples (sRNA) were stained with ethidium bromide after size separation by agarose gel electrophoresis and are shown below the main panels.

The results described above indicated that the *ago-1* gene product is the only Ago protein involved in RNA silencing triggered by exogenous transgenes during the vegetative growth, probably by binding the siRNAs produced by *M. circinelloides* Dcl-2. To analyze the presence of siRNAs in the *ago-1* ^−^ mutant, the small RNA fraction was isolated from the silenced wild type strain and ten non-silenced *ago-1* ^−^ mutants harbouring the *carB*-hpRNA expressing transgene and was hybridized with a *carB* antisense-specific riboprobe. RNA silencing in *M. circinelloides* is associated with the accumulation of two size classes of siRNAs, about 21 nt and 25 nt long [7]. These two classes were clearly detected in the silenced wild type strain, although no signal of siRNAs was detected in any of the 10 *ago-1* ^−^ mutants analyzed (Fig. 3). The same negative results were obtained when using for detection of siRNAs a method that improves the sensitivity 20- to 50-fold compared with standard methods [16] (results not shown), suggesting that the presence of a functional Ago-1 protein is essential for the accumulation of the Dcl-2-derived siRNAs. This could be due to the requirement of Ago-1 for the biogenesis or stability of the siRNAs, probably mediated by its interaction with other silencing proteins within a protein complex. We have investigated the interaction between Ago-1 and Dcl-2 using the yeast two hybrid system, although we could not detect any signal of specific interaction; similar negative results were obtained when using a modified system that allows detection of weak interactions. This lack of interaction could not be attributed to the absence of the fusion proteins, since they were detected in the yeast transformants by Western blot analysis using anti-AD or anti-BD antibodies (data not shown). The low efficiency of integrative transformation in *M. circinelloides* to tag the Ago-1 protein and the reduced specific activity of anti-Dcl-2 and anti-Ago-1 antibodies in immunoprecipitation assays has hampered the analysis of protein interactions in *M. circinelloides*.

### *ago-1* is Required for Accumulation of Endogenous ex-siRNAs

**Figure 4:**
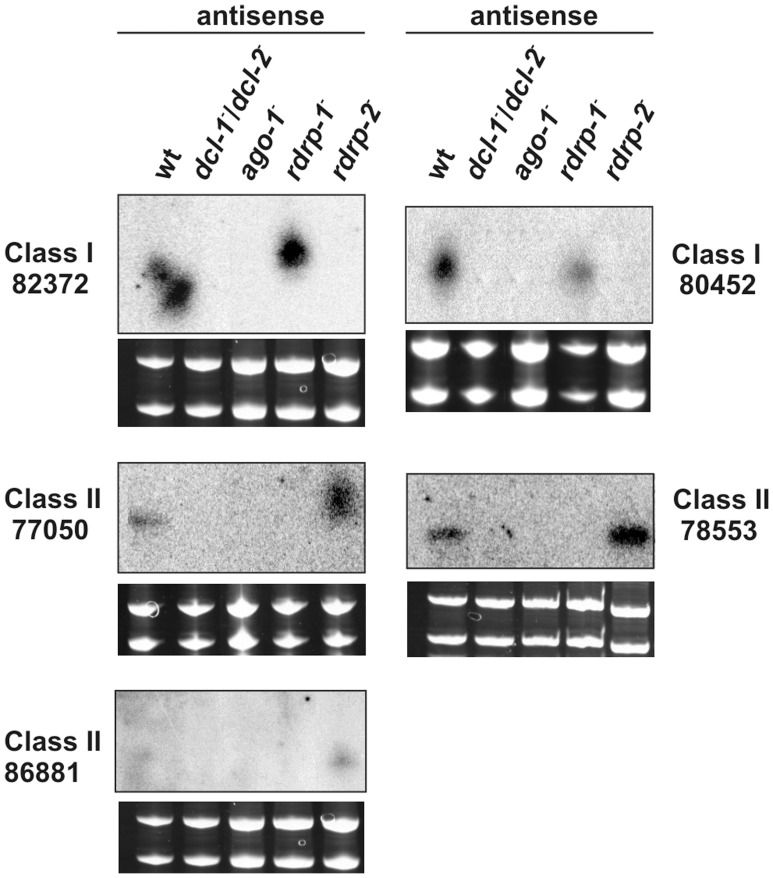
Accumulation of ex-siRNAs in wild type and silencing mutant *M. circinelloides* strains. Low-molecular weight RNA was extracted from wild-type, *dcl-1^−/^dcl-2* ^−^ double mutant, *ago-1* ^−^, *rdrp-1* ^−^ and *rdrp-2* ^−^ strains, separated on 15% denaturing polyacrylamide gels, transferred to membranes and probed with end-labelled DNA antisense-specific oligonucleotides specific to each locus. For exact probe sequences, see Table S4. Ethidium bromide stained images of gels below the radiograms show equal loading of lanes. The exon loci correspond to the following proteins: ID 82372, no domains found; ID 80452, serine/threonine kinase; ID 77050, no domains found; ID 78553, low similarity to transposase 21 protein; ID 86881, no domains found. Ten picomoles per lane of 23-mer to 27-mer DNA oligonucleotides in antisense orientation were used as size markers. In all cases, the RNA probes hybridized to these controls.

We have previously demonstrated that the *rdrp-1* and, at a minor extent, *rdrp-2* genes, are required for the production of different classes of endogenous siRNAs (esRNAs) in *M. circinelloides*, which are produced with the involvement of a Dicer activity [11]. Many of these esRNAs derive from exons (ex-siRNAs), although transposons-derived esRNAs were also detected. Three hundred and twenty four ex-siRNA loci were identified and classified in four classes based on the silencing proteins required for their biogenesis. Classes II and III, including 222 and 88 ex-siRNA-producing exons, respectively, are the largest ones [11]. To investigate the involvement of Ago-1 in the accumulation of the different classes of ex-siRNAs we analyzed the *M. circinelloides* sRNA content in the *ago-1* ^−^ mutant by deep sequencing of short RNAs (18–25 nt) and compared the *ago-1* ^−^ ex-siRNA profile with the wild type strain (accession number SRA SRR836082). The normalized reads of ex-siRNAs from the 324 exonic loci in the wild type and the *ago-1* ^−^ mutant strains are shown in Table S2, which also shows the coordinates of the ex-siRNA loci and the strand bias (sense/antisense) of the ex-siRNAs. The accumulation of ex-siRNAs from each exon in the *ago-1* ^−^ mutant strain was compared to the wild type and the fold difference is shown in Table S3, which includes data of the log_2_ fold change in the *dcl* ^−^ and *rdrp* ^−^ mutants [11]. The four classes of ex-siRNAs showed at least a 3-fold decrease in normalized reads in the *ago-1* ^−^ mutant compared to wild type (Table 3 and Table S3). All the class I and most of the class II (206 out of 222) ex-siRNAs were accumulated in a significantly lower amount in the *ago-1* ^−^ mutant relative to wild type (Fig. S4). The majority of the class II ex-siRNAs that were not down-regulated in the *ago-1* ^−^ mutant were just below the 3-fold threshold in this strain, showing a decrease of at least 2-fold (Table S3). Classes I and II of ex-siRNAs are *dcl-2* dependent, suggesting that *ago-1* is required for the accumulation of Dcl-2-derived endogenous sRNAs, whether or not these sRNAs require the RdRP-1 protein for their biogenesis. To confirm the involvement of Ago-1 in the accumulation of ex-siRNAs of classes I and II, northern blot experiments were carried out with selected loci of these classes (Fig. 4). In all cases, the presence of bands corresponding to 23–24 nt antisense siRNAs was confirmed in the wild type strain, while these bands were absent in the *ago-1* ^−^ mutant as well as in mutants affected in silencing genes previously known to be required for the biogenesis of these ex-siRNAs [11].

**Table 3:** Accumulation of the different classes of ex-siRNAs in the *ago-1* ^−^ mutant.

sRNA class	No. of exons	Down-regulated in	Average log_2_ fold change *ago-1* ^−^ vs WT^a^
Class I	9	*dcl-2* ^−^	−9.11
Class II	222	*dcl-2* ^−^, *rdrp-1* ^−^	−3.21
Class III	88	*dcl-1^−/^dcl-2* ^−^, *rdrp-1* ^−^, *rdrp-2* ^−^	−9.89
Class IV	5	*dcl-1* ^−^, *rdrp-1* ^−^, *rdrp-2* ^−^	−6.58

a Average value of the log_2_ fold change of the different classes of ex-siRNAs in the *ago-1* ^−^ *M. circinelloides* mutant compared to wild type. Log_2_ fold changes in Table S3 were used to calculate the average. Only values higher than 3-fold down-regulation in the corresponding mutants relative to wild type are indicated. Dependence on *dicer* and *rdrp* genes of ex-siRNAs from each class is shown.

Classes III and IV of ex-siRNAs were also down-regulated in the *ago-1* ^−^ mutant (Table 3 and Table S3). Eighty six out of 88 class III ex-siRNAs showed a marked decrease in the *ago-1* ^−^ mutant and four out of five ex-siRNA loci that corresponded to the tiny class IV were also down-regulated in this strain (Fig. S4). Besides the participation of different combinations of silencing proteins in their biogenesis, previous results indicated that class III ex-siRNAs displayed different features relative to those of classes I and II, such as a random spread of size distribution and a very strong strand bias, almost all of them being exclusively sense to the mRNAs [11]. This suggests that class III is not generated by a canonical silencing mechanism, although the ex-siRNA profiles obtained in the *ago-1* ^−^ mutant indicated that Ago-1 is required for the accumulation of class III ex-siRNAs.

### Ago-1 Specifically Binds Classes I and II of ex-siRNAs to Suppress Expression of Target Genes

The fact that all classes of ex-siRNAs are down-regulated in the *ago-1* ^−^ mutant strain could indicate that Ago-1 non-specifically binds all small RNAs and stabilises them, so removing Ago-1 drastically reduces the number of ex-siRNAs sequenced. To investigate the binding of Ago-1 to the different classes of ex-siRNAs, proteins from cleared extracts of the wild type strain and *ago-1* ^−^ mutant, which was used as negative control, were separated using a Superdex 200 10/300 size-exclusion column. The individual fractions were analyzed by western blot using anti-Ago-1 specific antibodies (Fig. S5) and sRNAs were isolated from the Ago-1-containing fractions and used to construct the cDNA library, once the presence of particular siRNAs in the Ago-1 containing fractions was confirmed (Fig. S5). Deep sequencing of the sRNAs bound to Ago-1 in the wild type strain and those isolated from equivalent fractions of the *ago-1* ^−^ mutant (accession number SRA SRR835448) identified a total of 417 siRNA loci that showed at least a 3-fold increase in normalized reads in the Ago-1 fraction purified from the wild type compared to the *ago-1* ^−^ mutant (Table S5). A high proportion of these loci corresponded to ex-siRNAs of classes I and II, whereas only two out of the 88 ex-siRNAs of class III and none of the class IV were detected among the Ago-1 bound siRNAs (Table 4 and Table S6). Ago-1 bound ex-siRNAs showed a strong preference for uridine at the 5′ position, a feature that is not shared with classes III and IV of ex-siRNAs, which preferentially contain a 5′ terminal adenosine (Fig. S6). These results suggest that ex-siRNAs from classes I and II are functional siRNAs and bind to Ago-1, whereas ex-siRNAs of classes III and IV require Ago-1 for their production but are not specifically bound to it.

**Table 4:** Binding of the different classes of ex-siRNAs to the Ago-1 protein in *M. circinelloides*.

sRNA class	No. of exons	Average log_2_ fold change Ago-1bound WT vs *ago-1* ^−a^	% of Ago-1 bound ex-siRNA loci
Class I	9	+8.31	100
Class II	222	+6.40	88.74 (197 out of 222)
Class III	88	–	2.27 (2 out of 88)
Class IV	5	–	0

a Log_2_ fold changes in Table S6 were used to calculate the average of the fold changes of Ago-1 bound ex-siRNAs in the WT compared with the *ago-1* ^−^ mutant. Only values higher than 3-fold up-regulation in the wild type relative to the *ago-1* ^−^ mutant are indicated.

**Figure 5:**
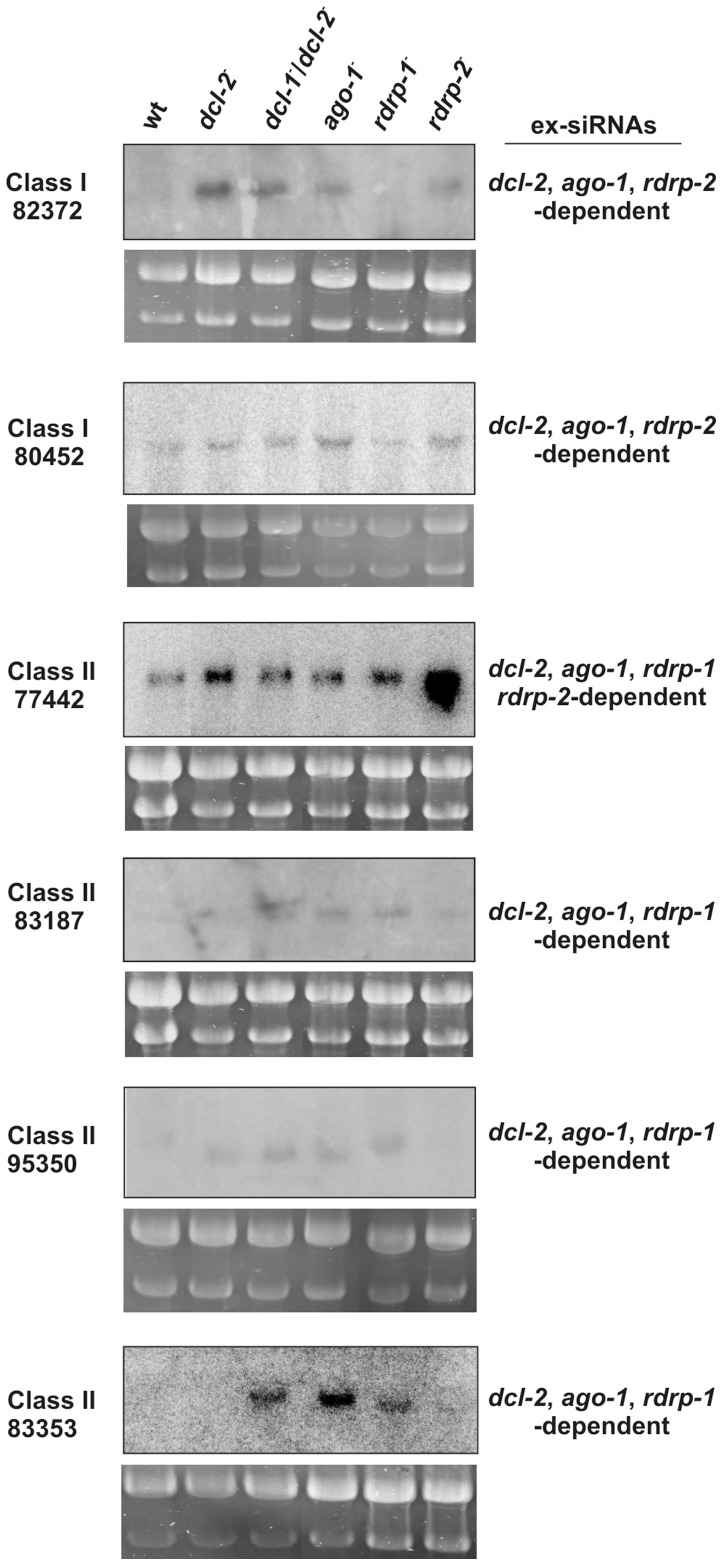
Accumulation of mRNAs in wild type and silencing mutant *M. circinelloides* strains. Northern blots of high molecular weight RNAs corresponding to ex-siRNA loci of classes I and II. Total RNA (50 µg) extracted from wild-type and mutant strains were separated in 1.2% denaturing agarose gel, transferred to membranes and hybridized with gene specific probes (Table S4). The exon loci correspond to the following proteins: ID 82372, no domains found; ID 80452, serine/threonine kinase; ID 77442, no domains found; ID 83187, chromodomain-helicase DNA-binding protein; ID 95350, no domains found; ID 83353, no domains found. Silencing genes involved in the biogenesis of the corresponding ex-siRNAs are indicated.

To confirm that *ago-1* participates in the regulation of endogenous genes through ex-siRNAs of classes I and II, accumulation of target mRNAs of selected loci was analyzed by Northern blot experiments. All the tested mRNA corresponding to loci that showed reduced ex-siRNA expression in the *ago-1* ^−^ mutant accumulated at an increased level in the *ago-1* ^−^ mutant strain compared to the wild type, as they did in the silencing mutants affected in genes required for the biogenesis of classes I and II ex-siRNAs (Fig. 5). Thus, reduction in ex-siRNAs accumulation results in an increase of mRNAs of the target genes. These results confirm that Ago-1 bound ex-siRNAs are functional, suppressing the expression of the corresponding target genes, and highlight the role of this protein in the regulation of expression of endogenous genes.

### *ago-1* ^-^ Mutant is Affected in Asexual Sporulation

**Figure 6:**
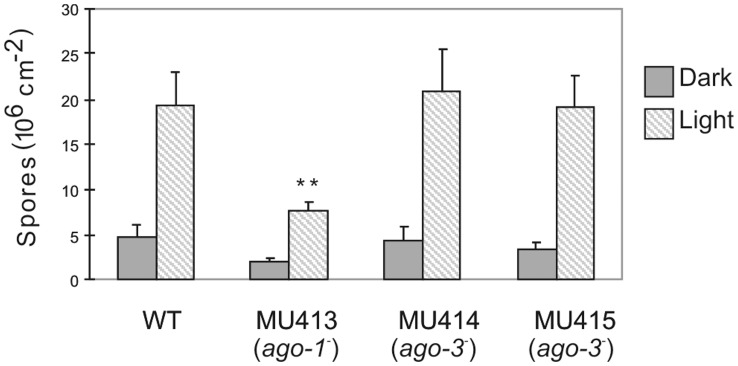
Effect of *ago-1* ^−^ mutation on asexual-spore production in *M. circinelloides*. Production of asexual spores in mycelia of the wild-type strain R7B, the *ago-1* ^−^ mutant MU413 and the *ago-3* ^−^ mutants MU414 and MU415 grown under white light and dark conditions for 3 days. The values are means and standard errors of 10 independent measurements.

The role of *ago-1* in the regulation of endogenous genes prompted us to perform a phenotypic analysis of the *ago-1* ^−^ mutant during vegetative growth. While its growth rate and hyphal morphology were similar to the wild type strain, a reduction in vegetative spore production was repeatedly detected in the *ago-1* ^−^ mutant. To quantify this phenotype, spores of the MU413 (*ago-1* ^−^), MU414 (*ago-3* ^−^), MU415 (*ago-3* ^−^) and wild-type mycelia (*n = *10) grown on MMC plates under dark or light conditions were harvested and counted (Fig. 6). Sporulation in the *ago* ^−^ mutants was stimulated by light, as occurs in the wild-type strain [17]. However, the spore production of the *ago-1^-^* mutant was significantly reduced relative to the wild-type strain, since there was less than 0.001 probability (Student *t* test) that the average spore production of the *ago-1* ^−^ mutant and that of the wild-type strain would be identical. This result suggests that *ago-1* is involved in the regulation of asexual development, probably through its role in the modulation of mRNA accumulation by endogenous silencing. This effect is specific of the *ago-1* gene, since the production of spores in the other *ago* ^−^ mutants was indistinguishable to that of the wild type strain (Fig. 6).

### *ago-1* ^-^ Mutant Shows Accelerated Autolysis under Nutritional Starvation

**Figure 7:**
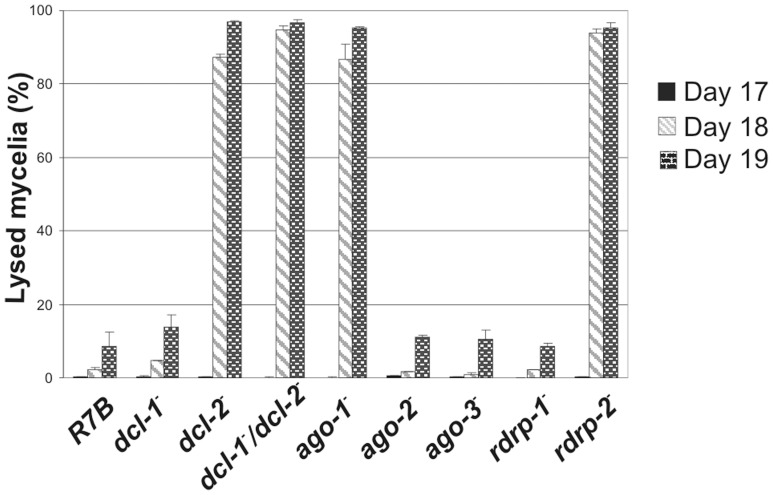
Autolysis in wild type and silencing mutant *M. circinelloides* strains. Percentages of lysed mycelia relative to the total mycelial area of the wild type R7B strain and mutants affected in silencing genes. Strains were grown in rich YPD medium under dark conditions from spores inoculated in the centre of Petri dishes until mycelia covered the complete plate area (2–3 days). After different incubation times, the lysed area of each mycelium relative to the total mycelial area was measured. The values are mean and standard errors of four independent measurements.

During the phenotypic analysis of the *ago-1* ^−^ mutant we realized that aged mycelia of that strain grown on solid YPD medium lysed much faster that the wild type strain (Fig. S7). Fungal autolysis is the natural process of self-digestion of aged hyphal cultures induced by nutrient starvation. Autolysis has been shown to be central to carbon-starving fungal cultures, with the last step of this process being degradation of the hyphal cell walls [18]. In fact, the accelerated autolysis phenotype shown by the *ago-1* ^−^ mutant is associated with the complete degradation of the fungal hyphae in the lysed area (Fig. S8). To quantify the early autolysis phenotype of the *ago-1* ^−^ mutant and extend this analysis to other silencing mutants, we grow mycelia of the wild type and different silencing mutant strains in solid YPD plates (n = 4) for different time periods and measured the lysed area of each mycelium at different intervals (see Materials and Methods). Results indicated that *ago-1* ^−^ mutant, as well as mutants affected in the *dcl-2* and *rdrp-2* genes, activated the lysis program induced by nutrient starvation at early time relative to the wild type strain, provoking thus the complete lysis of the mycelia within 24 hours once autolysis is initiated (Fig. 7 and Fig. S7). This phenotype is specific for the above mutants, since mutants affected in the *ago-2*, *ago-3* or *rdrp-1* genes did not show the accelerated autolysis phenotype, maintaining viable hypha for more than 30 days. This suggests that deficiency in some of the ex-siRNAs which are specifically dependent on *ago-1*, *dcl-2* and *rdrp-2* genes alters the lysis program activated by nutritional stress.

## Discussion

Besides a role in genome defence and genomic surveillance, knowledge on the regulatory functions of RNAi in fungi is limited to the construction of pericentric heterochromatin in *Schizosaccharomyces pombe* [19]. Several classes of esRNAs have been identified in different fungi, although their biological role remains to be determined due, in part, to the lack of phenotype of mutants affected in the RNAi machinery [4]. We demonstrated in this work that *M. circinelloides ago-1* is required for silencing exogenous sequences and for the production of regulatory esRNAs that derive from exons and regulate the expression of protein encoding genes. We also demonstrated that deletion of *ago-1* affects asexual development and autolysis, suggesting that genes involved in these processes are regulated by the RNAi machinery in this basal fungus.

The number of *argonaute* genes identified in filamentous fungal genomes range from zero to as many as eight [20]. The zygomycete *M. circinelloides* encodes three *ago* genes, although only *ago-1* participates in the RNAi machinery during vegetative growth (Table 1 and Table 2). Neither *ago-3* nor *ago-2* are involved in vegetative gene silencing and the corresponding null mutants do not have recognizable mutant phenotypes, although we cannot discard a role for those genes at different developmental stage or specialized tissues. Phylogenetic relationships of the *M. circinelloides* Ago proteins suggest that, although all of them belong to the AGO sub-family, they derive from different ancestors, being Ago-1 the most closely related to the Ago proteins of other zygomycetes (Fig. S1). All *M. circinelloides* Ago proteins contain the three negatively charged, evolutionarily conserved amino acids of the PIWI domain, as well as other conserved residues of the PAZ and MID domains (Fig. 1). The presence of all the conserved residues in Ago-2 is particularly striking, since it contains one in frame stop codon that would produce a truncated protein, which could indicate that the mutation has occurred recently. This pseudogene expresses at significant levels during vegetative growth (Fig. 2), although attempts to detect a truncated Ago-2 protein in Western blot experiments with specific anti-Ago-2 antibodies have been unsuccessful (data not show).

A dsRNA-induced expression of central genes of the RNAi pathway has been described in *N. crassa* as a part of a broad dsRNA response [21]. However, as it was previously demonstrated for *dcl-2* [9], expression of *ago-1* gene in *M. circinelloides* did not change by accumulation of dsRNAs, which could be explained if those genes have a constitutive expression pattern as a consequence of their role in the control of endogenous functions. Unexpectedly, *ago-1* expression, as well as *ago-2*, was significantly stimulated by light with an expression pattern that mimics other light-regulated genes in filamentous fungi (Fig. 2) ([22] and references therein). Light regulates many aspects of fungal biology, including development of reproductive structures and biosynthesis of carotenoids which improve adaptation and survival in the wild. The RNAi pathway in *M. circinelloides* seems to be mainly involved in the response to environmental signals such as those activating vegetative development or autolysis. Thus, it is tempting to speculate that light regulation of *ago-1* expression is part of a response aimed to modulate expression of light-responsive genes by the RNAi pathway. In fact, preliminary analysis indicates that a relevant number of ex-siRNAs derive from genes that are regulated by light through the action of the CrgA repressor and the White-Collar-1 homolog Mcwc-1b [23] (V. Garre, personal communication). Furthermore, the silencing efficiency in a *crgA* ^−^ mutant increases nearly 30-fold compared to the *crgA^+^* background [24], suggesting that activation of the light response provokes an activation of the RNAi machinery. Experiments are in progress to deepen into this pioneering relationship between light and RNAi regulation.

Ago-1 is required for transgene-induced gene silencing whatever the nature of the silencing trigger is, as shown by the negative silencing phenotype of *ago-1* ^−^ mutants expressing sense- or inverted-repeat transgenes (Table 1). We have not been able to detect primary or secondary siRNAs in those mutants, even though several sensitive assays were used (Fig. 3), suggesting that Ago-1 is required for production/stability of siRNAs. Most of siRNAs detected in *M. circinelloides* silenced strains corresponds to secondary siRNAs derived from target transcripts [7]. Thus, lack of accumulation of siRNAs in *ago-1* ^−^ mutants could be due, at least in part, to a role of Ago-1 in the biogenesis of secondary siRNAs, as occurs in metazoans. In plants, production of secondary siRNAs requires the slicer activity of Ago proteins on mRNA targets to provide templates for RdRP amplification, whereas in *C. elegans* AGO-primary siRNA complexes are required for recruiting RdRPs to target transcripts [3]. Alternatively, lack of accumulation of siRNAs in *ago-1* ^−^ mutants could indicate that these small RNAs are stabilized by binding to Ago-1, so that in its absence the siRNAs would be rapidly degraded.

Besides a role in RNA silencing induced by exogenous sequences, different classes of Ago proteins are required for the production of a wide repertoire of esRNA molecules in higher eukaryotes [25]. In mammals, these molecules include miRNAs that regulate the expression of protein coding genes, endo-siRNAs that mostly map to intergenic repetitive elements and pseudogenes and piRNAs, which silence transposons. In *Drosophila melanogaster*, different Ago proteins associate to miRNAs, endo-siRNAs, piRNAs and exo-siRNAs derived from viral sequences and a similar situation is found in plants [25]. We have demonstrated that in the basal fungus *M. circinelloides* a single Ago protein, Ago-1, is required both for silencing exogenous sequences and endogenous protein coding genes during vegetative development, suggesting that the functional diversification of Ago proteins occurred after the separation of the fungal kingdom. Among *M. circinelloides* esRNAs, classes I and II correspond to siRNAs derived from exons that regulate the expression of the protein coding genes from which they derive [11]. These two classes, which are Dcl-2-dependent, are down-regulated in the *ago-1* ^−^ mutant, suggesting that Ago-1 is involved in the biogenesis/stability of these regulatory ex-siRNAs (Table S2 and Table S3). In fact, validation experiments demonstrated that lack of detection of ex-siRNAs in the *ago-1* ^−^ mutant is associated with an increase of mRNA accumulation of the target genes (Fig. 4 and Fig. 5). These two classes of ex-siRNAs specifically bind to Ago-1 (Table S5 and Table S6), which indicates that they are functional siRNAs produced by a canonical RNAi pathway to suppress the expression of the corresponding target genes. The Ago-1 bound ex-siRNAs showed a very strong bias for uracil at the 5′ position (Fig. S6). The 5′ end of small RNAs is a crucial factor for various functional aspects of RNA silencing. Analysis of small RNAs associated with AGO proteins in *Arabidopsis thaliana* identified a strong preference for a particular 5′ nucleotide in the small RNA to interact with specific members of the AGO protein family [26], based on the nucleotide specificity loop of the MID domain. The preference for 5′ uracil of *M. circinelloides* Ago-1 may explain lack of binding of class III ex-siRNAs, which show a preference for adenine at the 5′ end (Fig. S6).

As mentioned before, the down-regulation of classes I and II ex-siRNAs in the *ago-1* ^−^ mutant could be explained by a role of Ago in the biogenesis and/or stability of the sRNAs. This is not the case of ex-siRNAs of classes III and IV. These sRNAs do not specifically bind Ago-1, at least tight enough to be isolated as an RNA-protein complex. Thus, the down-regulation of these ex-siRNAs in the *ago-1* ^−^ mutant can’t be explained by stabilization of these sRNAs by Ago binding but it could suggest that classes III and IV ex-siRNAs require Ago-1 for their production, as it has been recently demonstrated for some milRNAs in *N. crassa* [27]. In fact, the *N. crassa* Ago protein QDE-2 is absolutely required for mil-R1 biogenesis in a mechanism that is independent of its slicer activity but involves interaction with exosome and other silencing proteins. Lack of binding to Ago-1 also indicates that classes III and IV ex-siRNAs are not *bona fide* ex-siRNAs but rather they might act through a non canonical RNA silencing mechanism, a frequent situation in filamentous fungi [28]. Alternatively, classes III and IV ex-siRNAs may bind to a different Ago protein. The low level of expression of the *ago-3* gene during the vegetative cycle rules out the possibility of analyzing binding of classes III and IV ex-siRNAs to Ago-3, since several specific antibodies anti-Ago-3 failed to detect this protein in western blot experiments (data not shown). The biogenesis and structural characteristics of class III ex-siRNAs are unusual. They do not show the strong bias for uracil at the 5′ position observed in Ago-1 bound ex-siRNAs (Fig. S6) and they show a random spread of size distribution that is not observed in the ex-siRNAs of classes I and II, which produce predominantly 23–24 nt sRNAs [11]. Furthermore, almost all class III ex-siRNAs are exclusively sense to the mRNAs. It has been proposed that these ex-siRNAs are produced by the polymerase activity of *M. circinelloides* RdRP proteins that generate dsRNA stretches from target mRNAs, and the dicing activity of either Dcl-1 or Dcl-2 on these discrete dsRNA regions. After the initial cleavage, the single stranded portions of mRNAs are degraded by non-specific RNases [11]. Results obtained in this work indicate that, besides Dicer and RdRP proteins, Ago-1 is required for the production of class III ex-siRNAs, possibly by participating in a multiprotein complex that signals to an unidentified ribonuclease the mRNAs that has to be degraded.

The phenotypic characteristics of *ago-1* ^−^ mutant suggest that it is affected in the response to environmental signals. The relationship between endogenous regulation by RNAi and environmental conditions has been recently demonstrated in the fission yeast *S. pombe*, where RNAi and heterochromatin factors cooperate to silence loci that are regulated by environmental and developmental signals, such as genes up-regulated during sexual differentiation [29]. Generation of siRNAs and heterochromatin modifications at specific loci were also observed in carbon-limiting conditions or at low temperature, indicating that heterochromatin domains formed by RNAi are regulated by physiological conditions. In *S. pombe*, both the 3′-5′ exonuclease activity of the exosome and RNAi are important to fully silence these target loci [29], which results in mild or no defect in single mutants. The reduction in the production of asexual spores in *ago-1* ^−^ mutants (Fig. 6) could indicate a predominant role of RNAi in the regulation of this developmental process in which many genes are involved [17], [30]. Besides a defect in sporulation, several silencing mutants, including *ago-1* ^−^, share a common phenotype regarding to the autolytic response to nutritional stress (Fig. 7, Fig. S7 and Fig. S8). Fungal autolysis is the physiological process of self-digestion of aged hyphal cultures, occurring as a result of hydrolase activity, causing vacuolation, formation of empty hyphae and disruption of organelle and cell wall structure [31]. The autolysis of filamentous fungi is not a simple cell necrosis phenomenon but is an active and well regulated process where many enzymatic activities are involved [18]. The accelerated lysis phenotype shown by *ago-1* ^−^, *dcl-2* ^−^ and *rdrp-2* ^−^ mutants suggests that this process could be controlled by gene/s regulated by ex-siRNAs. Some of the class I ex-siRNAs require *ago-1*, *dcl-2* and *rdrp-2* for their biogenesis (locus 82372; Fig. 4 and Table S3) and the corresponding target transcript shows an increased accumulation in *ago-1* ^−^, *dcl-2* ^−^ and *rdrp-2* ^−^ mutants (Fig. 5). Locus 82372 encodes a calcium/calmodulin-dependent protein kinase of the EF-Hand superfamily of calcium-binding proteins that shows significant similarity to cell-cycle checkpoint protein kinases of the Cds1/Rad53/ChK2 family (maximum E value 2e^−102^, maximum identity 29%) (protein ID 109224 in the version 2 of the *M. circinelloides* genome: http://genome.jgi-psf.org/Mucci2/Mucci2.home.html). These proteins activate in response to DNA damage to prevent entry into mitosis, leading to cell cycle arrest in G1 [32]. This protein family has been also implicated in linking the cell cycle to diverse processes such as senescence and the circadian cycle [33]. It is tempting to speculate that alteration of the protein levels in *M. circinelloides* silencing mutants could affect the signalling pathway, accelerating a DNA damage-induced cell death in long-lived cells. Future studies may reveal the specific role of this and other proteins regulated by ex-siRNAs in the response to environmental signals in *M. circinelloides*.

### Conclusions

The Argonaute family in *M. circinelloides* is composed by three members, although only one, Ago-1, is required for the production of transgene-induced siRNAs and the accumulation of four different classes of regulatory esRNAs during vegetative growth, suggesting that the functional diversification of Ago proteins found in metazoans occurred after the separation of the basal lineage of fungi. The different classes of esRNAs, which are generated through different pathways, bind differentially to Ago-1, revealing the complexity of the biogenesis pathways in this basal fungus. According with the key role of Ago-1 in endogenous silencing, deletion of *ago-1* affects asexual development and autolysis, strongly suggesting that RNAi in this basal fungus silences genes regulated by environmental signals.

## Materials and Methods

### Strains, Growth and Transformation Conditions

The leucine auxotroph R7B, derived from *M. circinelloides f. lusitanicus* CBS 277.49 (syn. *Mucor racemosus* ATCC 1216b) was used as the wild-type strain. Strain MU402 [8], an uracil and leucine auxotroph derived from R7B, was used as the recipient strain in transformation experiments to knock out *ago* genes. Strain MU406 is a *dcl-1* ^−^ null mutant derived from MU402 [8]. Strain MU410 is a *dcl-2* ^−^ null mutant derived from MU402 [9]. Strain MU411 is a double *dcl-1^−/^dcl-2* ^−^ null mutant derived from MU410 [9]. Strains MU419 and MU420 correspond to *rdrp-1* ^−^ and *rdrp-2* ^−^ null mutants derived from MU402, respectively [10]. Cultures were grown at 26°C in minimal YNB medium, complete YPG medium or MMC medium [8]. When indicated, complete yeast and dextrose agar (YPD) media was used. Media were supplemented with uridine (200 µg/ml) or leucine (20 µg/ml) when required. The pH was adjusted to 4.5 or 3.2 for mycelial or colonial growth respectively. Transformation was carried out as previously described [7]. Transformants were grown in selective medium for several vegetative cycles to increase the proportion of transformed nuclei, as primary transformants are heterokaryons due to the presence of several nuclei in the protoplasts. Illumination conditions were as previously described [34]. *Escherichia coli* strain DH5α was used for all cloning experiments and strain LE392 (Promega, Madison, WI) for the propagation of the *M. circinelloides* genomic lambda clones.

### Plasmids

A complete description of plasmids used in this work for cloning and functional analysis of the *ago* genes can be found in Materials and Methods S1.

### Nucleic Acid Manipulation and Analysis

Standard recombinant DNA manipulations were performed as described [35]. Genomic DNA of *M. circinelloides* was prepared as previously reported [8]. To identify transformants which correctly integrated the knockout vectors designed to disrupt the *ago* genes, a rapid protocol for isolation of DNA to be used in PCR amplifications was utilized [8]. Total RNA was isolated using Trizol reagent, following the recommendations of the supplier (Invitrogen). Southern blot and Northern blot hybridizations were carried out under stringent conditions [8].

DNA probes were labelled with [α-^32^P]dCTP using Ready-To-Go Labelling Beads (GE Healthcare Life Science). For Northern blot experiments, *ago*-specific probes corresponding to the 5′-terminal regions of the three *ago* genes were used. The *ago-1* probe corresponds to a 370 bp fragment amplified with primers argo28 and argo29 (Table S1). The *ago-2* probe corresponds to an 820 bp fragment obtained by PCR amplification with primers argo13 and argo27 (Table S1). The *ago-3* probe corresponds to a 266 bp fragment amplified with primers argo30 and argo31 (Table S1). The percentages of identity among the three probes ranged from 58 to 64%, and their specificity was confirmed by Northern blot experiments (Fig. S2). Signal intensities were estimated from autoradiograms using a Shimadzu CS-9000 densitometer.

For Southern blot experiments, specific *ago* probes that discriminate between the wild type and disrupted alleles were obtained for each *ago* genes. Probe *a* corresponds to a 900 bp fragment of the *ago-1* gene amplified with primers argo23 and argo24 (Table S1), and probe *b* corresponds to the same 370 bp fragment used in Northern experiments. For *ago-2* gene, probe *c* corresponding to a 400 bp fragment amplified with primers argo13 and argo38 (Table S1) and probe *d* corresponding to an 840 bp *Nco*I fragment isolated from plasmid pMAT1334 were obtained. Probe *e* corresponds to a 1.2 kb *Bam*HI-*Sac*I fragment of the *ago-3* gene isolated from plasmid pMAT1305 and probe *f* corresponds to the same 266 bp fragment used in Northern experiments.

Expand Reverse Transcriptase (Roche) and PfuUltra Hotstart DNA Polymerase (Stratagene) or EcoTaq Plus (Ecogen, Spain) were used in PCR and RT-PCR experiments. RT-PCR amplifications were performed to confirm introns in the *ago-1* and *ago-2* genomic sequences. Total RNA was reverse transcribed with specific primers for each *ago* gene. Primers pairs argo29 and argo34, and argo32 and argo33 (Table S1) were used to identify exon-intron boundaries in *ago-1* gene. Primers pairs argo4 and argo13, and argo6 and argo13 (Table S1) were used to identify introns in *ago-2* gene. The amplified fragments were cloned in the *Eco*RV site of pBluescript II SK+ vector and sequenced.

### Cloning of *Ago* Genes

In order to clone the *M. circinelloides argonaute* homologous genes, degenerated primers corresponding to conserved regions of the known Argonaute proteins were designed and used in PCR experiments with *M. circinelloides* genomic DNA as a template. To reduce the degree of degeneration, sequences of the two *argonaute*-like genes of *Rhizopus oryzae*, a zygomycete closely related to *M. circinelloides* whose genomic sequence was available (http://www.broadinstitute.org/annotation/genome/rhizopus_oryzae/MultiHome.html), were considered as references. Two degenerated oligonucleotide primers were designed to amplify the *ago*-like genes of *M. circinelloides*. Sense primer argosense3 (5'-CAAACTGC(CT)GA(AG)ATGAT(ACT)AA(AG)TT-3') corresponds to the sequence QTAEMIKF of the PAZ domain and the antisense primer argoantis3 (5'-AATTGGCC(CT)TC(AG)CT(AGCT)AC(AGCT)CC(AG)TC-3') corresponds to the sequence DGVSEGQF of the PIWI domain. Genomic PCR amplifications were carried out at 50°C using genomic DNA of the R7B as a template. Amplification with these primers gave rise to a 966 bp fragment that was sequenced after being cloned into pGEM-T Easy vector (Promega, Madison, WI). Analysis of the deduced amino acid sequence revealed a truncated ORF with a very high degree of similarity to the corresponding fragment of the *R. oryzae ago* genes (95.4% with RO3G_13047.3 [*ago1*] and 91.6% with RO3G_10137.3 [*ago2*]). The *M. circinelloides* gene was named *ago-1*. To clone the genomic version of this gene, as well as other putative *ago* genes present in the genome of *M. circinelloides*, 10,000 plaques from a genomic LambdaGEM-11 library of wild-type CBS 277.49 strain [34] were transferred to a Colony/Plaque Screen™ membrane (NEN, Ma) and screened with the 966 pb fragment of the *ago-1* gene (*ago* probe in Fig. 1A). Hybridization to the radioactively labelled probe was carried out at 65°C under high-stringency conditions [8]. Eight hybridizing clones were isolated and their DNAs analyzed by restriction and Southern analyses. As a result, the clones were divided into three different groups. Hybridizing fragments from a representative clone of each group were subcloned into the pBluescript II SK+ vector giving rise to plasmids pMAT1301, pMAT1333 and pMAT1305 (Materials and Methods S1). Sequence analysis of pMAT1301 identified a 5′-truncated genomic version of the *ago-1* gene. The complete version of this gene was cloned by chromosomal walking, using inverse PCR. Plasmids pMAT1333 and pMAT1305 contained the complete versions of two different ORFs sharing high similarities to *ago-1*, which were named *ago-2* and *ago-3* genes, respectively.

### Sporulation Measurements

To quantify the sporulation efficiencies of *ago* mutants, spores were harvested from *ago* ^−^ and wild-type mycelia as described previously [17]. Briefly, small pieces of mycelium (2-mm diameter; *n* = 10) of the different strains were transplanted to MMC solid medium, pH 3.2, and grown for 72 h under dark or light conditions. After incubation, the diameter of each colony was determined, and the complete colony was harvested in 1.0 ml distilled water and vortexed to allow suspension of the spores. The spores were counted with a hemocytometer.

### Lysis Measurements

Five hundred spores of each strain were inoculated in the centre of YPD plates (n = 4) and incubated at 26°C under dark conditions for 2–3 days, until mycelia covered the complete plate area. Incubation was maintained at the same conditions for longer time and autolysis of aged mycelia was estimated by image analysis of the plates. Images were acquired using a scanner (HP Scanjet G4010). Lysis estimation was performed using ImageJ, an open source image analysis program (*rsbweb.nih.gov/ij/*). Images were transformed from RGB to 8-bit mode. Mycelial area and autolysis area were selected separately by thresholding their gray levels (8-bit images provide 256 values of gray [0–255], 0 = black, 255 = white). Gray levels 1–112 were ranged for lysis and levels 107–255 ranged for mycelium. The obtained binary masks were measured and the results were expressed as percent of autolysis (lysis area/total area of the plate).

### FPLC Size-exclusion Chromatography and Western Blot Analysis

Mycelia of the different strains grown for 48 hours in complete YPG medium were frozen in liquid nitrogen and triturated by mortar and pestle. The cell powder was suspended in extraction buffer (20 mM Tris-HCl pH 7.5, 300 mM NaCl, 5 mM MgCl_2_ and 5 mM DTT) containing 1∶50 dilution of the Protease Inhibitor Cocktail (Sigma P-8215) and 1 mM benzamidina. The lysate was kept on ice for 30 min and then twice centrifuged at 10,000 *g* at 4°C and filtered through a 0.2 µm Millipore filter to produce a cleared lysate. Cleared extracts were directly loaded on the Superdex 200 (10/300) column, which was equilibrated with extraction buffer. Chromatography was performed at 0.25 ml/min, collecting 500 µl fractions. Proteins from individual fractions were precipitated with 20% TCA, washed with acetone and suspended in NuPAGE LDS Sample buffer (Invitrogen). Proteins were separated by electrophoresis in 4–12% acrylamide gels (NuPAGE Novex Bis-Tris Mini Gels, Invitrogen) and electrotransferred to nitrocellulose membrane (Protan, Whatman). Membranes were incubated with monospecific anti-*M. circinellides* Ago-1 antibodies, which were generated against a 18 amino acid Ago-1-specific peptide (Invitrogen). The specificity of the anti-Ago-1 antibody was validated by Western blot analysis of the *ago-1* ^−^ mutant. Inmunocomplexes were detected by the chemiluminescent ECL system (ECL Western Blotting Detection System, Amersham). Signals were detected using the Typhoon 9410 scanner (GE Healthcare).

### Small RNA Analysis

For siRNA analysis, low molecular weight RNA was prepared from total RNA, separated by electrophoresis and hybridized to radioactive riboprobes as previously described [9]. When indicated, the crosslinking to the Hybond-NX® membrane (Amersham) was carried out with l-ethyl-3-(3-dimethylaminopropyl) carbodiimide (EDC) (Sigma) as described [16]. EDC-cross-linking improves the sensitivity of detection of siRNAs compared with standard UV-cross-linking procedures by 20–50 times [16]. The *carB* antisense-specific riboprobe was prepared by *in vitro* transcription from linearized pMAT652 plasmid [7], using the MAXIscript transcription kit (Ambion). Oligonucleotides corresponding to sense and antisense sequences of the *carB* gene were used as size and polarity controls.

For endogenous sRNA analysis, small RNA samples were extracted from mycelia grown for 48 h on YPG plates as described above. cDNA libraries of small RNAs from the wild type and *ago-1* ^−^ mutant strains were generated and sequenced as described previously [11]. For isolation of sRNAs bound to the Ago-1 protein, small RNA samples were extracted from Ago-1-containing FPLC fractions using Trizol reagent. The 20–25 nt long sRNAs were gel purified and used to construct the cDNA library as described previously [11]. Equivalent fractions from the *ago-1* ^−^ mutant were used for isolation of sRNAs as a negative control. Sequencing of the Ago-1 bound cDNA libraries were carried out on an Illumina Genome Analyzer IIα with 50 nt read length (Baseclear). For detection of endogenous sRNAs in Northern blot experiments, membranes were hybridized overnight at 37°C in ULTRAhybOligo hybridization buffer (Ambion). DNA oligonucleotide probes were labelled with [gamma-^32^P]-ATP using T4 polynucleotide kinase (Promega). Membranes were exposed to phosphorimager screens (Fujifilm) and scanned on a Molecular Imager FX reader (BioRad).

### Sequence Analysis

Sequences analyses were carried out using European Bioinformatic Institute Server software (EMBL Outstation, Hinxton, UK), the National Center for Biotechnology Information Server (NCBI, Bethesda, MD, USA), ExPASy Molecular Biology Server (Swiss Institute of Bioinformatics) and Baylor College of Medicine Search Launcher (Houston, TX).

For analysis of endogenous sRNAs, the raw reads were processed and normalized as previously described [11]. sRNAs were mapped to the *M. circinelloides* genome, whose sequence was available from the JGI (http://genome.jgi-psf.org/Mucci1/Mucci1.home.html) (v 1.0), using PatMaN [36]. Reads mapping in close proximity (≤200 bp) to each other were grouped together into loci [11] and mapped to annotated exons, transposons and intergenic regions. Only 21–24 nucleotide reads matching the reference sequence were retained for subsequent analysis in the Ago-1 bound sRNAs samples. sRNA loci were said to be down-regulated in the *ago-1* ^−^ sample if the normalised locus abundance showed at least a threefold decrease in comparison to the wild type sample (log_2_ fold change ≥ −1.6). sRNAs were said to be bound to Ago-1 if the normalized abundance in the Ago-1 fractions purified from the wild type strain showed at least a threefold increase relative to the *ago-1* ^−^ sample (log_2_ fold change ≥1.6). To increase the stringency of the analysis and avoid lowly expressed regions, any loci with a normalised abundance count of less than 50 in the wild type were excluded from the analysis.

### Nucleotide Sequences

The nucleotide sequences of the *ago* genes are now available at http://genome.jgi-psf.org/Mucci2/Mucci2.home.html (v 2.0). The location of the *ago-1* sequence in the *M. circinelloides* genomic sequence is Mucci2/scaffold_01:3735839–3738600 and the protein ID 104161. The location of the *ago-2* sequence is Mucci2/scaffold_09:522961–525659 and the protein ID 195366. The location of the *ago-3* sequence is Mucci2/scaffold_06:1878601–1881328 and the protein ID 104163. No other *ago*-like sequences have been identified in the *M. circinelloides* genome sequence.

The raw reads of *M. circinelloides* small RNAs have been deposited in the Sequence Read Archive (SRA) database under the accession numbers SRR836082 (small RNAs in *ago-1* ^−^ mutant) and SRR835448 (Ago-1-bound small RNAs in wild type and *ago-1* ^−^ mutant).

## Supporting Information

Figure S1 **Argonaute proteins of Zygomycetes. A.** Identity and similarity (in parentheses) between the deduced amino acid sequences of *M. circinelloides* Ago proteins and similar proteins of the zygomycetes *Rhizopus oryzae* and *Phycomyces blakeskeeanus*. The sequences used in the alignment were: *R. oryzae* RO3G_13047.3 (Ago1) and RO3G_10137.3 (Ago2) and *P. blakesleeanus* 123569 (AgoA) and 85795 (AgoB). *P. blakesleeanus* sequences are available at http://genome.jgi-psf.org/Phybl2/Phybl2.home.html. **B.** Phylogenetic relationship of *M. circinelloides* Ago proteins (McAgo-1, McAgo-2 and McAgo-3) and similar proteins of the zygomycetes *R. oryzae* (RoAgo1 and RoAgo2) and *P. blakeskeeanus* (PbAgoA and PbAgoB). *A. thaliana* Ago-1 (NP_849784) was used as external sequence. Phylogenetic tree was constructed using PhyML 3.0 aLRT method (maximum likelihood) [37] from sequence alignment created using MUSCLE 3.7 [38], using the Phylogeny software (http://www.phylogeny.fr) [39]. Branch lengths are proportional to the number of substitutions per site (bars). The numbers at the nodes are bootstrap values (%) for 100 replications.
(PDF)

Figure S2 **Specificity of the** ***ago*** **probes.** Northern blot analysis of total RNA (50 µg) isolated from the wild type strain R7B and the *ago-1* ^−^, *ago-2* ^−^ and *ago-3* ^−^ mutants grown for 24 hours in liquid MMC medium. Filters were hybridized with *ago*-specific probes corresponding to the 5′ region of each *ago* gene, where they showed the maximum sequence divergence (see Materials and Methods) and reprobed with a 28S rRNA probe to check loading.
(TIF)

Figure S3 **Disruption of the** ***ago*** **genes. A.** Genomic structure of *ago-1* wild-type locus (middle) and after homologous recombination of the replacement fragment (below). Gray boxes, coding sequence of the *ago-1* gene; black boxes, genomic regions flanking the *ago-1* gene; white boxes, *pyrG* selectable marker. The positions of the probes used (probes *a* and *b*) and the expected sizes of the *Eco*RV and *Kpn*I restriction fragments detected by the probes are indicated. Primers pyrG10F and argo26 were used to identify integration events. E, *Eco*RV; K, *Kpn*I. **B.** Similar representation of the *ago-2* locus in the wild type and disrupted strains. The positions of the probes used (probes *c* and *d*) and the expected sizes of the *Hin*dIII-*Xho*I or *Hin*dIII restriction fragments detected by the probes are indicated. Primers pyrGZ and argo41 were used to identify integration events. H, *Hin*dIII; X, *Xho*I. **C.** Similar representation of the *ago-3* locus in the wild type and disrupted strains. The positions of the probes used and the expected sizes of *Pvu*II restriction fragments detected by the probes are indicated. Primers pyrGZ and argo37 were used to identify integration events. P, *Pvu*II. **D.** Southern blot analysis of the wild-type strain R7B and the *ago-1* ^−^ mutant MU413. Genomic DNA (1 µg) was digested with *Eco*RV or *Kpn*I and hybridized with probes *a* (left) and *b* (right) (Fig. S3A). The positions and sizes of the GeneRuler DNA ladder mixture (Fermentas) size markers are indicated. **E.** Southern blot analysis of the wild-type strain R7B and the *ago-2* ^−^ mutants MU416 and MU417. Genomic DNA (1 µg) was double-digested with *Hin*dIII and *Xho*I and hybridized with probes *c* (left) and *d* (right) (Fig. S3B). The positions and sizes of the GeneRuler DNA ladder mixture (Fermentas) size markers are indicated. **E.** Southern blot analysis of the wild-type strain R7B and the *ago-3* ^−^ mutants MU414 and MU415. Genomic DNA (1 µg) was digested with *Pvu*II and hybridized with probes *e* (left) and *f* (right) (Fig. S3C). The positions and sizes of the GeneRuler DNA ladder mixture (Fermentas) size markers are indicated.
(TIF)

Figure S4 ***ago-1*** **dependence of the different classes of ex-siRNAs.** The pie charts show the percentage of ex-siRNA loci of each class that show reduced level of sRNAs in the *ago-1* ^−^ mutant strain. Class I had been defined as *dcl-2* dependent and *rdrp-1* independent, although most of the ex-siRNAs of this class depend on *rdrp-2*, whereas class II is *dcl-2* and *rdrp-1* dependent. Class III requires both *rdrp-1* and *rdrp-2* but *dcl-1* and *dcl-2* have redundant roles in its biogenesis. Class IV requires *dcl-1* and the two *rdrp* genes.
(TIF)

Figure S5 **Purification of Ago-1-enriched sRNAs. A.** Elution profile obtained from the size-exclusion chromatography on a Superdex 200 (10/300) column of a cleared extract of the silenced wild type strain containing the *carB* inverted repeat transgene (pMAT1253). FPLC was performed at 0.25 ml/min, collecting 500 µl fractions. AU: arbitrary units. The individual fractions were analyzed by Western blot using anti-Ago-1 primary antibody. The specificity of the anti-Ago-1 antibody was confirmed by Western blot analysis of the three *ago* ^−^ knockout mutants (inset). Asterisk marks an unspecific band detected by the anti-Ago-1 antibody in all the strains tested. To confirm the presence of Ago-1-bound siRNAs, low molecular weight RNA was extracted from each fraction, separated on 15% denaturing polyacrylamide gels, transferred to membranes and probed with a *carB*-antisense specific riboprobe (pMAT652; [7]). The two size classes of *carB*-derived siRNAs were only detected in the Ago-1 containing fractions. One picomol per lane of 29-mer DNA oligonucleotides in antisense orientation (Ribo3) and 25-mer oligonucleotide in sense orientation (carB25) were used as controls. **B.** Western blot analysis of FPLC fractions obtained from cleared extract of the wild type strain (left) and an *ago-1* ^−^ mutant, which was used as negative control (right). Ago-1 containing fractions of the wild type strain were pooled and used for isolation of Ago-1 enriched sRNAs. The same fractions of the *ago-1* ^−^ mutant were used as negative control.
(TIF)

Figure S6 **Nucleotide preference at the 5′ end of ex-siRNAs.** The percentage of the four bases (thymidine/uracil, cytosine, guanine and adenine) at the 5′ end of small RNA reads was calculated for the ex-siRNAs bound to the Ago-1 protein in the wild type strain (**A**) and the class III ex-siRNAs (**B**). The composition of the whole exons is shown for comparison in **C**.
(TIF)

Figure S7 **Lysis in aged mycelia of the** ***ago-1*** ^−^ **strain and other silencing mutants.** Comparison of mycelial lysis of *M. circinelloides* among the wild type strain R7B, the *dicer* mutants MU406 (*dcl-1* ^−^), MU410 (*dcl-2* ^−^) and MU411 (*dcl-1^−/^dcl-2* ^−^), the *ago* mutants MU413 (*ago-1* ^−^), MU416 *(ago-2* ^−^) and MU414 (*ago-3* ^−^) and the *rdrp* mutants MU419 (*rdrp-1* ^−^) and MU420 (*rdrp-2* ^−^). Images were taken after incubation for 13, 17, 18 and 25 days in YPD plates.
(TIF)

Figure S8 **Formation of empty hyphae and hyphal degradation in aged mycelia of the** ***ago-1*** ^−^ **mutant. A**. Age mycelia of the *ago-1* ^−^ mutant show accelerated autolysis compared to the wild type strain. **B**. Lysed mycelia of *ago-1* ^−^ mutant present vacuolization and empty hypha in the border between the mycelial and autolysis areas (left), whereas the central part of the autolysis area is completely free of hyphal material (right). Cultures were grown for 18 days in YPD plates. Scale = 100 mm.
(TIF)

Table S1 **Sequences of oligonucleotides used for cloning and functional analysis of the** ***ago*** **genes.**
(PDF)

Table S2 **Normalized reads and strand bias of ex-siRNAs in the wild type and** ***ago-1*** ^−^ **mutant strains.** Normalized reads in the *ago-1* ^−^ mutant (*ago-1* ^−^ abundance) of ex-siRNAs corresponding to each locus is compared to wild type. The coordinates correspond to the exonic loci where small RNAs maps. Strand bias indicates orientation to mRNAs, where 1 corresponds to all sRNAs in the same orientation as the mRNA, 0 to equal mixture of sRNAs on both strands and −1 to all sRNAs antisense to mRNAs. The data are sorted by the transcripts ID numbers.
(XLS)

Table S3 **Log_2_ fold change of ex-siRNAs in different mutants compared to wild type.** Normalized reads in Table S2 were used to calculate the fold change of ex-siRNAs in the *ago-1* ^−^ mutant compared to wild type strain. Log_2_ of the fold changes in *dcl* ^−^ (*dcl-1* ^−^, *dcl-2* ^−^ and the double mutant *dcl-1^−/^dcl-2* ^−^) and *rdrp* ^−^ (*rdrp-1* ^−^ and *rdrp-2* ^−^) mutants are also shown in this table. The data are sorted for the fold change in *dcl-2* ^−^ strain, which almost perfectly separated the four ex-siRNA classes from each other. Class 1, 2, 3 and 4 exons have yellow, white, blue and grey background, respectively. Values that represent a threefold or larger change (Log_2_ fold ≥ −1.6) are in bold. Decrease in expression is shown in red and increase in expression is shown in green.
(XLS)

Table S4 **Oligonucleotides used in validation experiments.**
(PDF)

Table S5 **Normalized reads and log_2_ fold change of Ago-1-bound siRNAs in the wild type compared with the** ***ago-1*** ^−^ **mutant.** The data are sorted for the fold change value. Only loci with a normalized abundance count higher than 50 in the wild type strain and a log_2_ fold change ≥1.6 (threefold or larger change) were considered in the analysis.
(XLS)

Table S6 **Distribution of siRNAs bound to Ago-1 among the different classes of ex-siRNAs.** The fold change of Ago-1 bound siRNAs in the WT compared with the *ago-1* ^−^ mutant is shown for the ex-siRNA loci identified among the siRNAs bound to Ago-1. The log_2_ fold change of ex-siRNAs in the *ago-1* ^−^ mutant compared with the wild type strain is shown for comparison. Class 1, 2, 3 and 4 exons have yellow, white, blue and grey background, respectively. N/A: non detected ex-siRNAs.
(XLS)

Material and Methods S1 (PDF)
