# Supplementary material for: A Single Argonaute Gene Participates in Exogenous and Endogenous RNAi and Controls Cellular Functions in the Basal Fungus Mucor circinelloides
Source: PLoS One. 2013 Jul 23;8(7):e69283. doi: 10.1371/journal.pone.0069283 (PMC3720535; doi:10.1371/journal.pone.0069283)

Figure S1

A

	M. circinelloides Ago-1	*M. circinelloides* Ago-2	*M. circinelloides* Ago-3
M. circinelloides Ago-2	56,6 (67,3)	-	
M. circinelloides Ago-3	57,3 (74,0)	51,7 (73,3)	-
R. oryzae Ago1	**85,7** (93,5)	55,6 (72,6)	55,8 (73,5)
R. oryzae Ago2	**81,8** (90,4)	55,8 (72,9)	55,5 (72,6)
P. blakesleeanus AgoA	**78,9** (86,7)	53,9 (68,9)	51,4 (68,0)
P. blakesleeanus AgoB	**63,2** (76,9)	48,2 (67,3)	47,6 (67,3)

B

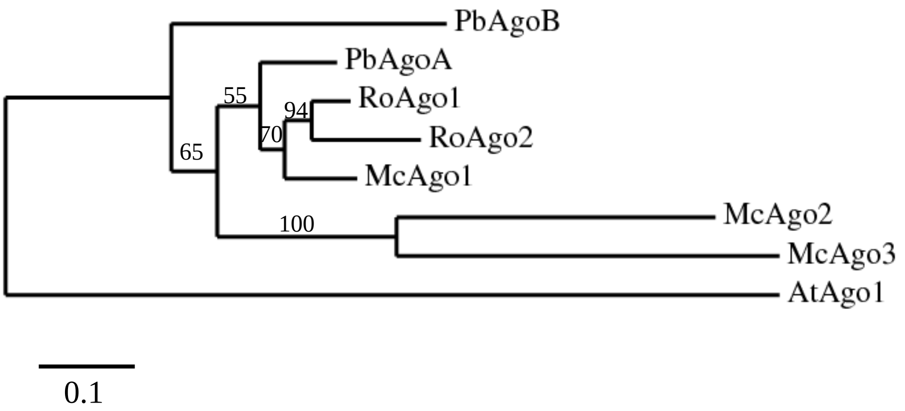

Supplement: Figure S1 — Argonaute proteins of Zygomycetes. A. Identity and similarity (in parentheses) between the deduced amino acid sequences of M. circinelloides Ago proteins and similar proteins of the zygomycetes Rhizopus oryzae and Phycomyces blakeskeeanus. The sequences used in the alignment were: R. oryzae RO3G_13047.3 (Ago1) and RO3G_10137.3 (Ago2) and P. blakesleeanus 123569 (AgoA) and 85795 (AgoB). P. blakesleeanus sequences are available at http://genome.jgi-psf.org/Phybl2/Phybl2.home.html. B. Phylogenetic relationship of M. circinelloides Ago proteins (McAgo-1, McAgo-2 and McAgo-3) and similar proteins of the zygomycetes R. oryzae (RoAgo1 and RoAgo2) and P. blakeskeeanus (PbAgoA and PbAgoB). A. thaliana Ago-1 (NP_849784) was used as external sequence. Phylogenetic tree was constructed using PhyML 3.0 aLRT method (maximum likelihood) [37] from sequence alignment created using MUSCLE 3.7 [38], using the Phylogeny software (http://www.phylogeny.fr) [39]. Branch lengths are proportional to the number of substitutions per site (bars). The numbers at the nodes are bootstrap values (%) for 100 replications. (PDF) [file pone.0069283.s001.pdf]
