# Supplementary figures and images for: A Single Argonaute Gene Participates in Exogenous and Endogenous RNAi and Controls Cellular Functions in the Basal Fungus Mucor circinelloides
Source: PLoS One. 2013 Jul 23;8(7):e69283. doi: 10.1371/journal.pone.0069283 (PMC3720535; doi:10.1371/journal.pone.0069283)

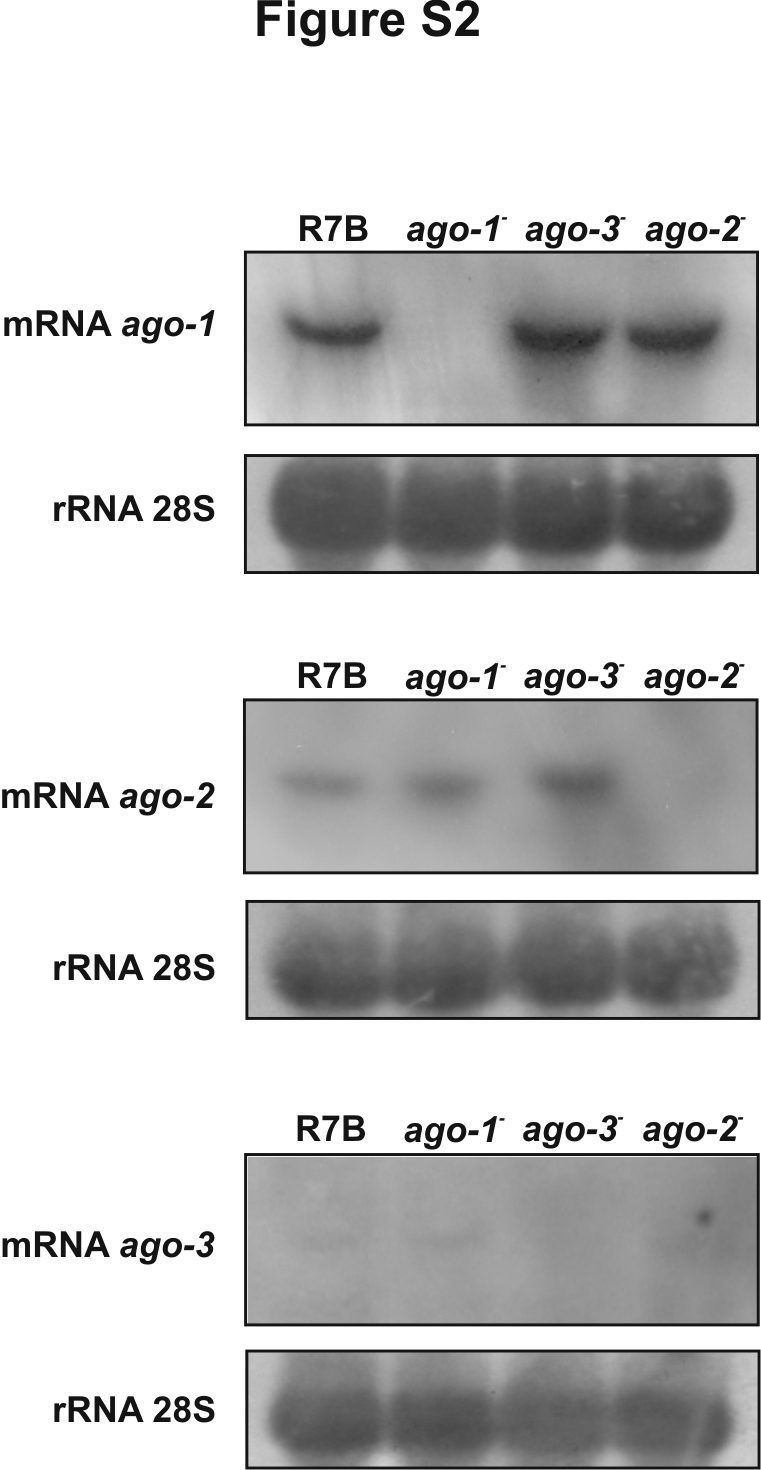

Supplement: Figure S2 — Specificity of the ago probes. Northern blot analysis of total RNA (50 µg) isolated from the wild type strain R7B and the ago-1 −, ago-2 − and ago-3 − mutants grown for 24 hours in liquid MMC medium. Filters were hybridized with ago-specific probes corresponding to the 5′ region of each ago gene, where they showed the maximum sequence divergence (see Materials and Methods) and reprobed with a 28S rRNA probe to check loading. (TIF) [file pone.0069283.s002.tif]

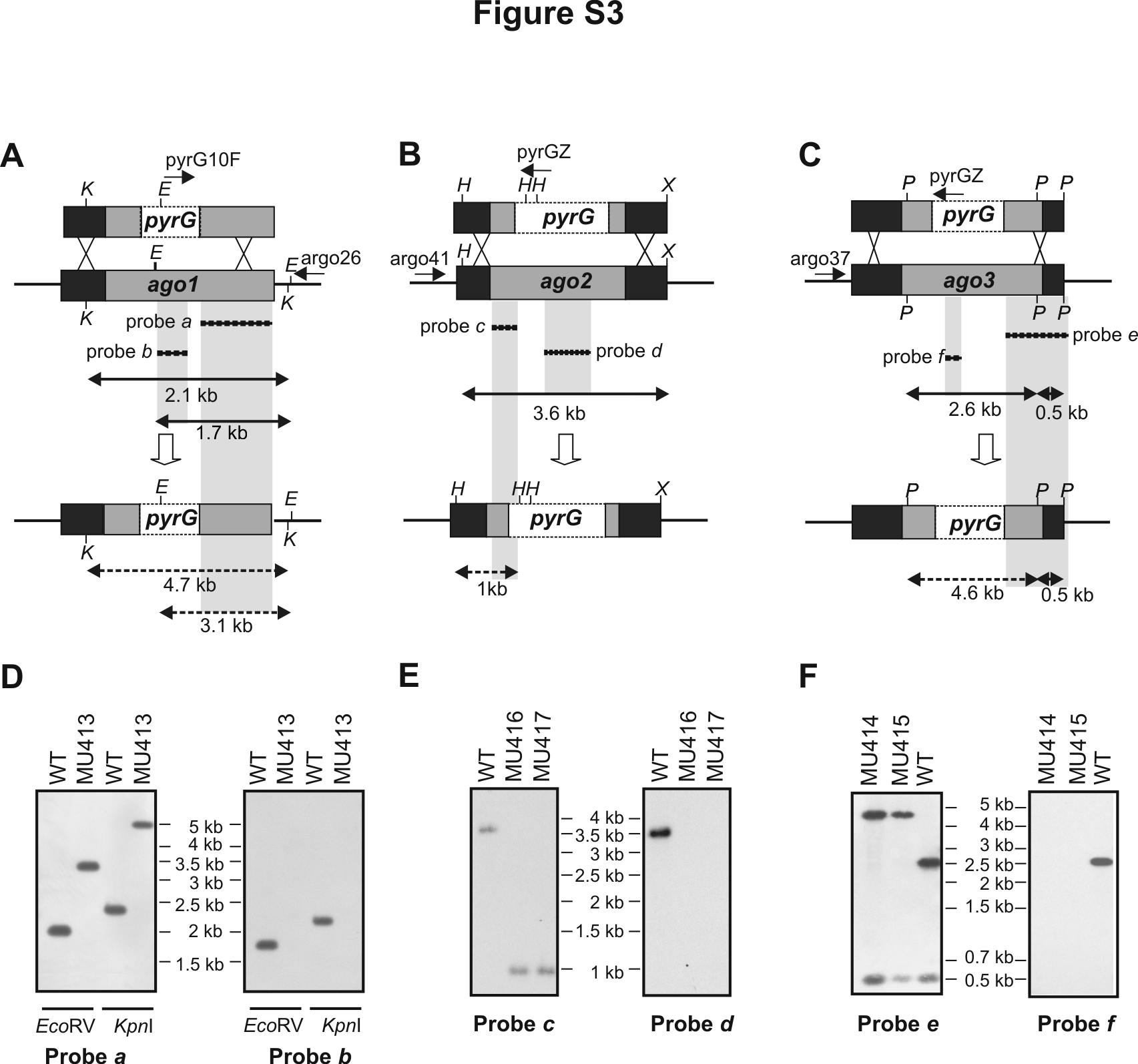

Supplement: Figure S3 — Disruption of the ago genes. A. Genomic structure of ago-1 wild-type locus (middle) and after homologous recombination of the replacement fragment (below). Gray boxes, coding sequence of the ago-1 gene; black boxes, genomic regions flanking the ago-1 gene; white boxes, pyrG selectable marker. The positions of the probes used (probes a and b) and the expected sizes of the EcoRV and KpnI restriction fragments detected by the probes are indicated. Primers pyrG10F and argo26 were used to identify integration events. E, EcoRV; K, KpnI. B. Similar representation of the ago-2 locus in the wild type and disrupted strains. The positions of the probes used (probes c and d) and the expected sizes of the HindIII-XhoI or HindIII restriction fragments detected by the probes are indicated. Primers pyrGZ and argo41 were used to identify integration events. H, HindIII; X, XhoI. C. Similar representation of the ago-3 locus in the wild type and disrupted strains. The positions of the probes used and the expected sizes of PvuII restriction fragments detected by the probes are indicated. Primers pyrGZ and argo37 were used to identify integration events. P, PvuII. D. Southern blot analysis of the wild-type strain R7B and the ago-1 − mutant MU413. Genomic DNA (1 µg) was digested with EcoRV or KpnI and hybridized with probes a (left) and b (right) (Fig. S3A). The positions and sizes of the GeneRuler DNA ladder mixture (Fermentas) size markers are indicated. E. Southern blot analysis of the wild-type strain R7B and the ago-2 − mutants MU416 and MU417. Genomic DNA (1 µg) was double-digested with HindIII and XhoI and hybridized with probes c (left) and d (right) (Fig. S3B). The positions and sizes of the GeneRuler DNA ladder mixture (Fermentas) size markers are indicated. E. Southern blot analysis of the wild-type strain R7B and the ago-3 − mutants MU414 and MU415. Genomic DNA (1 µg) was digested with PvuII and hybridized with probes e (left) and f (right) (Fig. S3C). The positions and sizes [file pone.0069283.s003.tif]

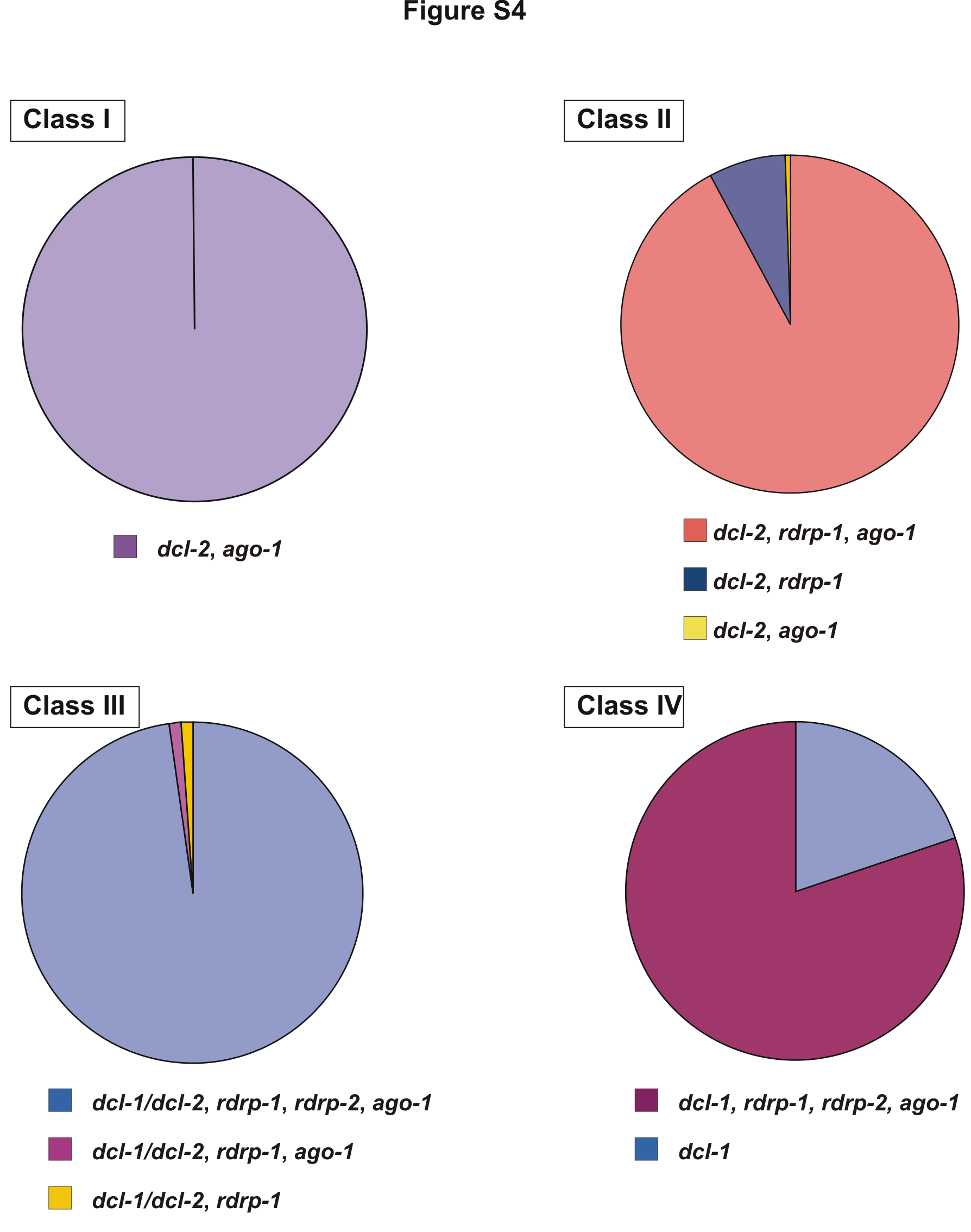

Supplement: Figure S4 — ago-1 dependence of the different classes of ex-siRNAs. The pie charts show the percentage of ex-siRNA loci of each class that show reduced level of sRNAs in the ago-1 − mutant strain. Class I had been defined as dcl-2 dependent and rdrp-1 independent, although most of the ex-siRNAs of this class depend on rdrp-2, whereas class II is dcl-2 and rdrp-1 dependent. Class III requires both rdrp-1 and rdrp-2 but dcl-1 and dcl-2 have redundant roles in its biogenesis. Class IV requires dcl-1 and the two rdrp genes. (TIF) [file pone.0069283.s004.tif]

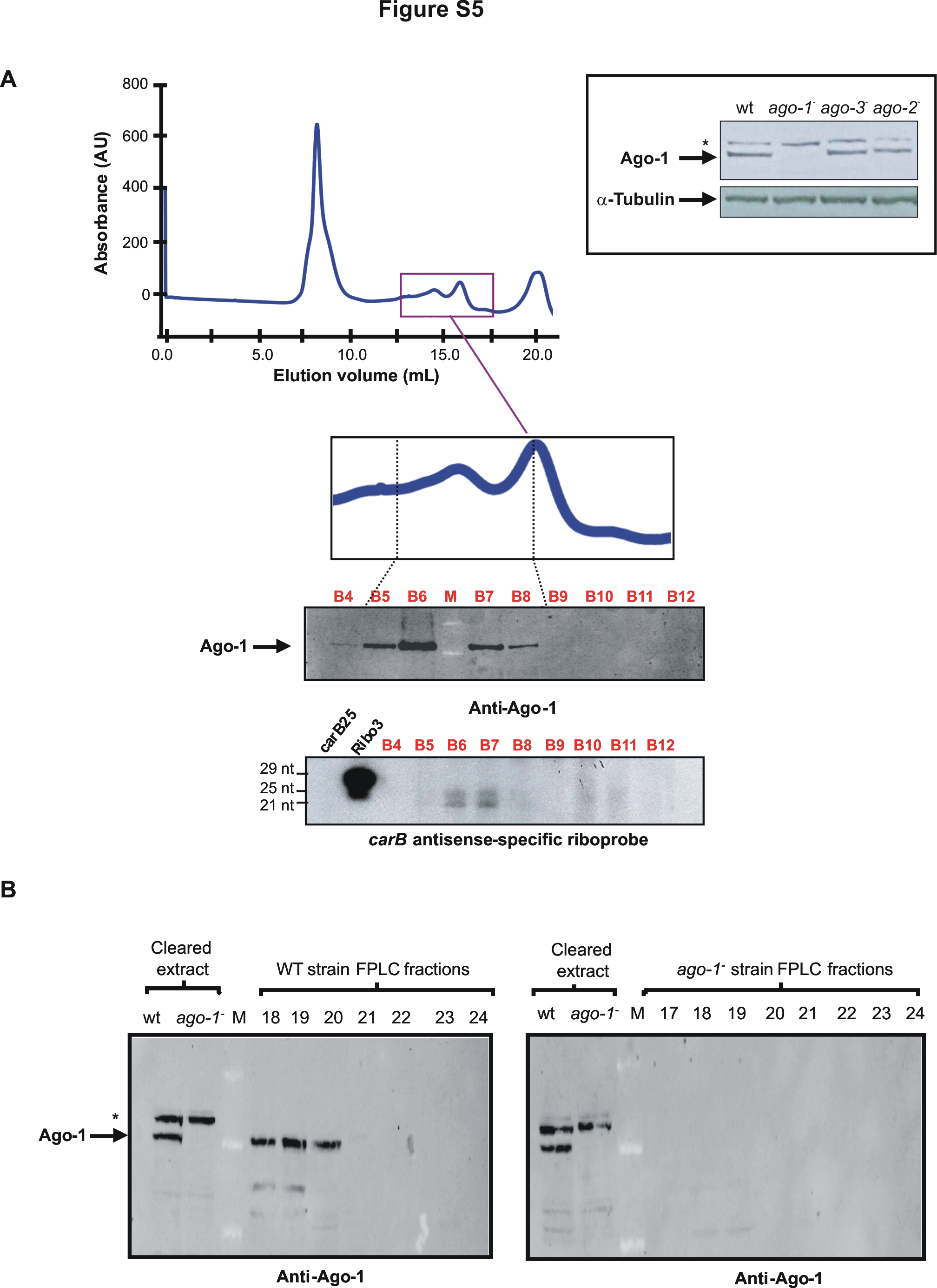

Supplement: Figure S5 — Purification of Ago-1-enriched sRNAs. A. Elution profile obtained from the size-exclusion chromatography on a Superdex 200 (10/300) column of a cleared extract of the silenced wild type strain containing the carB inverted repeat transgene (pMAT1253). FPLC was performed at 0.25 ml/min, collecting 500 µl fractions. AU: arbitrary units. The individual fractions were analyzed by Western blot using anti-Ago-1 primary antibody. The specificity of the anti-Ago-1 antibody was confirmed by Western blot analysis of the three ago − knockout mutants (inset). Asterisk marks an unspecific band detected by the anti-Ago-1 antibody in all the strains tested. To confirm the presence of Ago-1-bound siRNAs, low molecular weight RNA was extracted from each fraction, separated on 15% denaturing polyacrylamide gels, transferred to membranes and probed with a carB-antisense specific riboprobe (pMAT652; [7]). The two size classes of carB-derived siRNAs were only detected in the Ago-1 containing fractions. One picomol per lane of 29-mer DNA oligonucleotides in antisense orientation (Ribo3) and 25-mer oligonucleotide in sense orientation (carB25) were used as controls. B. Western blot analysis of FPLC fractions obtained from cleared extract of the wild type strain (left) and an ago-1 − mutant, which was used as negative control (right). Ago-1 containing fractions of the wild type strain were pooled and used for isolation of Ago-1 enriched sRNAs. The same fractions of the ago-1 − mutant were used as negative control. (TIF) [file pone.0069283.s005.tif]

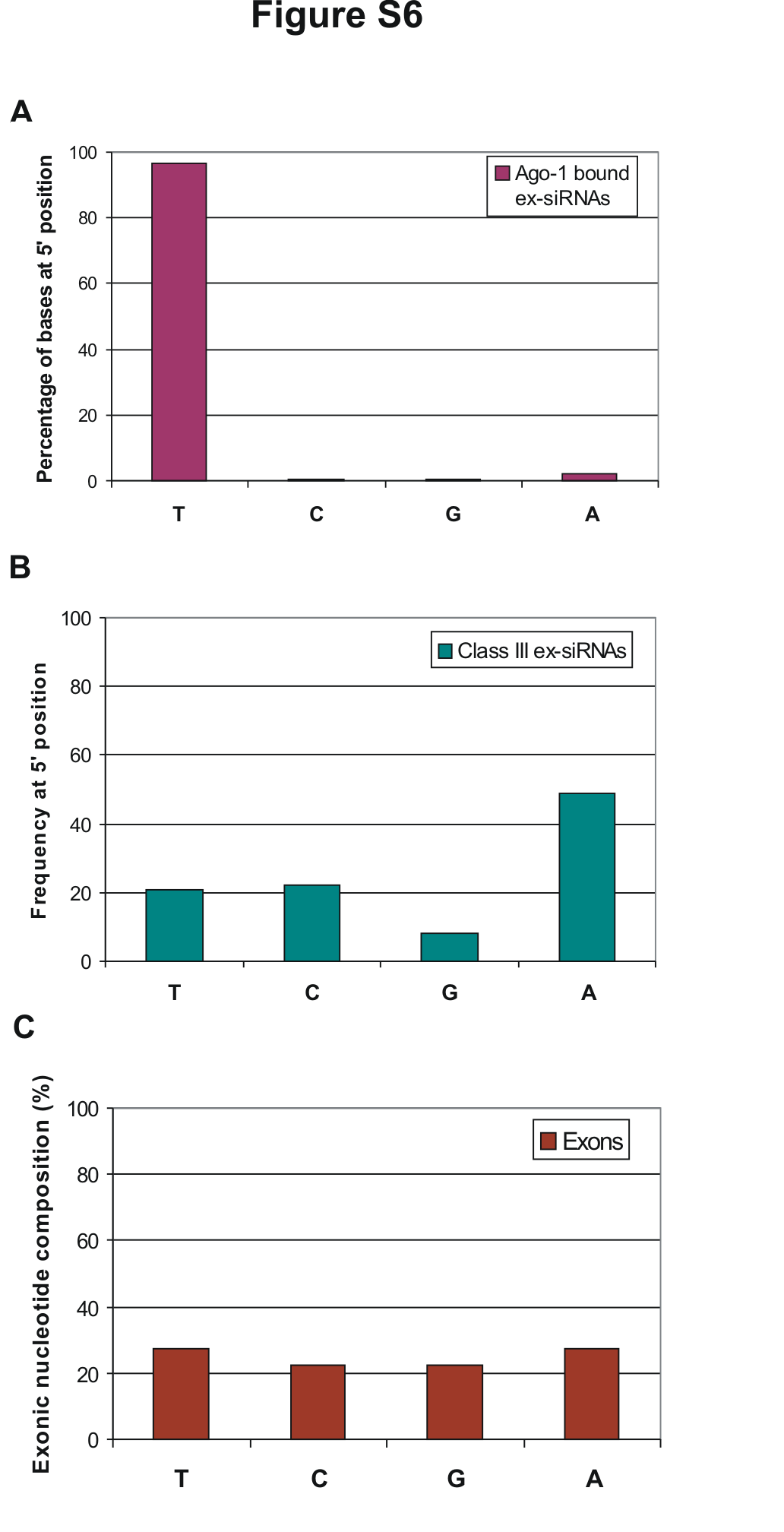

Supplement: Figure S6 — Nucleotide preference at the 5′ end of ex-siRNAs. The percentage of the four bases (thymidine/uracil, cytosine, guanine and adenine) at the 5′ end of small RNA reads was calculated for the ex-siRNAs bound to the Ago-1 protein in the wild type strain (A) and the class III ex-siRNAs (B). The composition of the whole exons is shown for comparison in C. (TIF) [file pone.0069283.s006.tif]

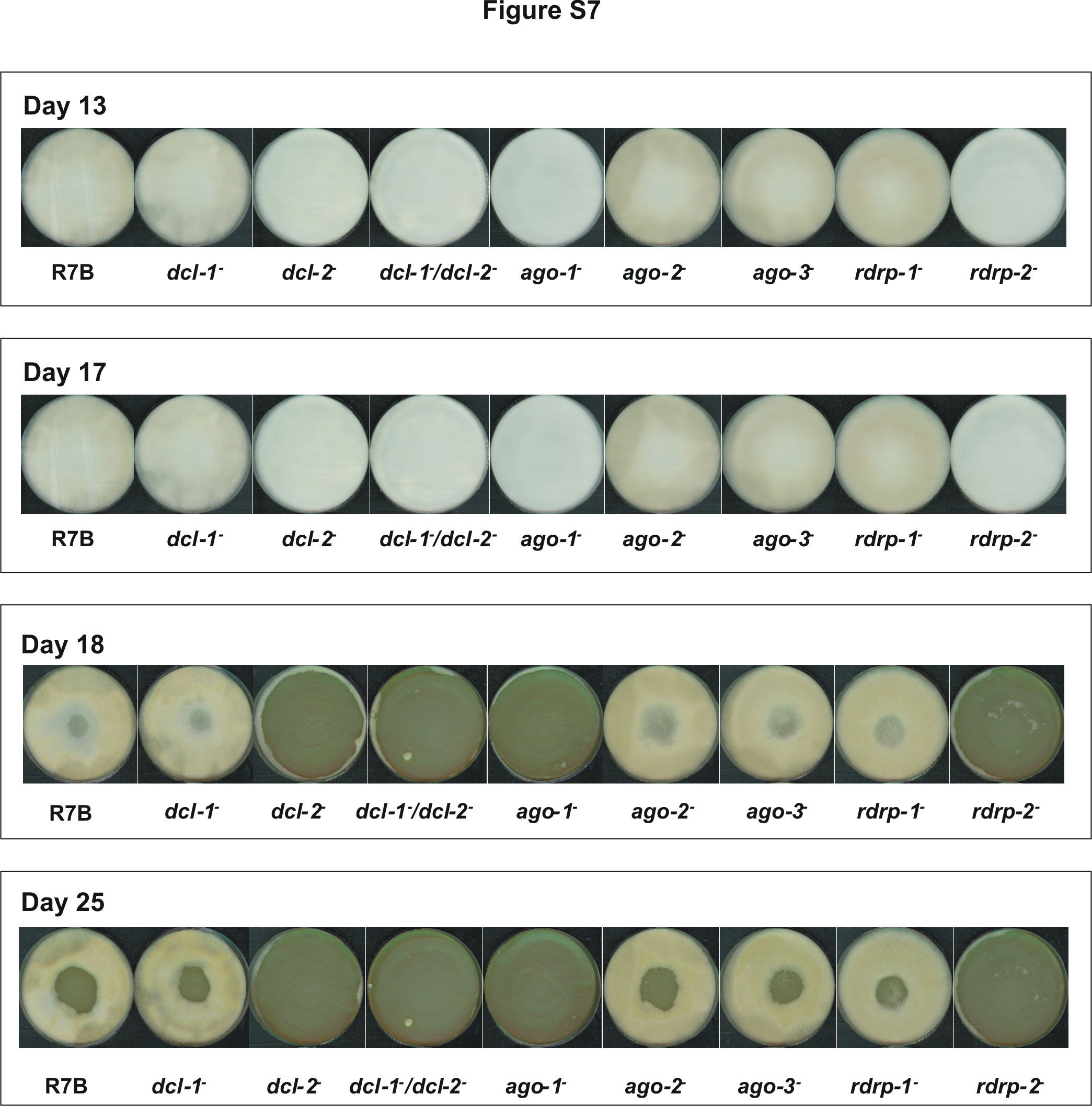

Supplement: Figure S7 — Lysis in aged mycelia of the ago-1 − strain and other silencing mutants. Comparison of mycelial lysis of M. circinelloides among the wild type strain R7B, the dicer mutants MU406 (dcl-1 −), MU410 (dcl-2 −) and MU411 (dcl-1−/dcl-2 −), the ago mutants MU413 (ago-1 −), MU416 (ago-2 −) and MU414 (ago-3 −) and the rdrp mutants MU419 (rdrp-1 −) and MU420 (rdrp-2 −). Images were taken after incubation for 13, 17, 18 and 25 days in YPD plates. (TIF) [file pone.0069283.s007.tif]

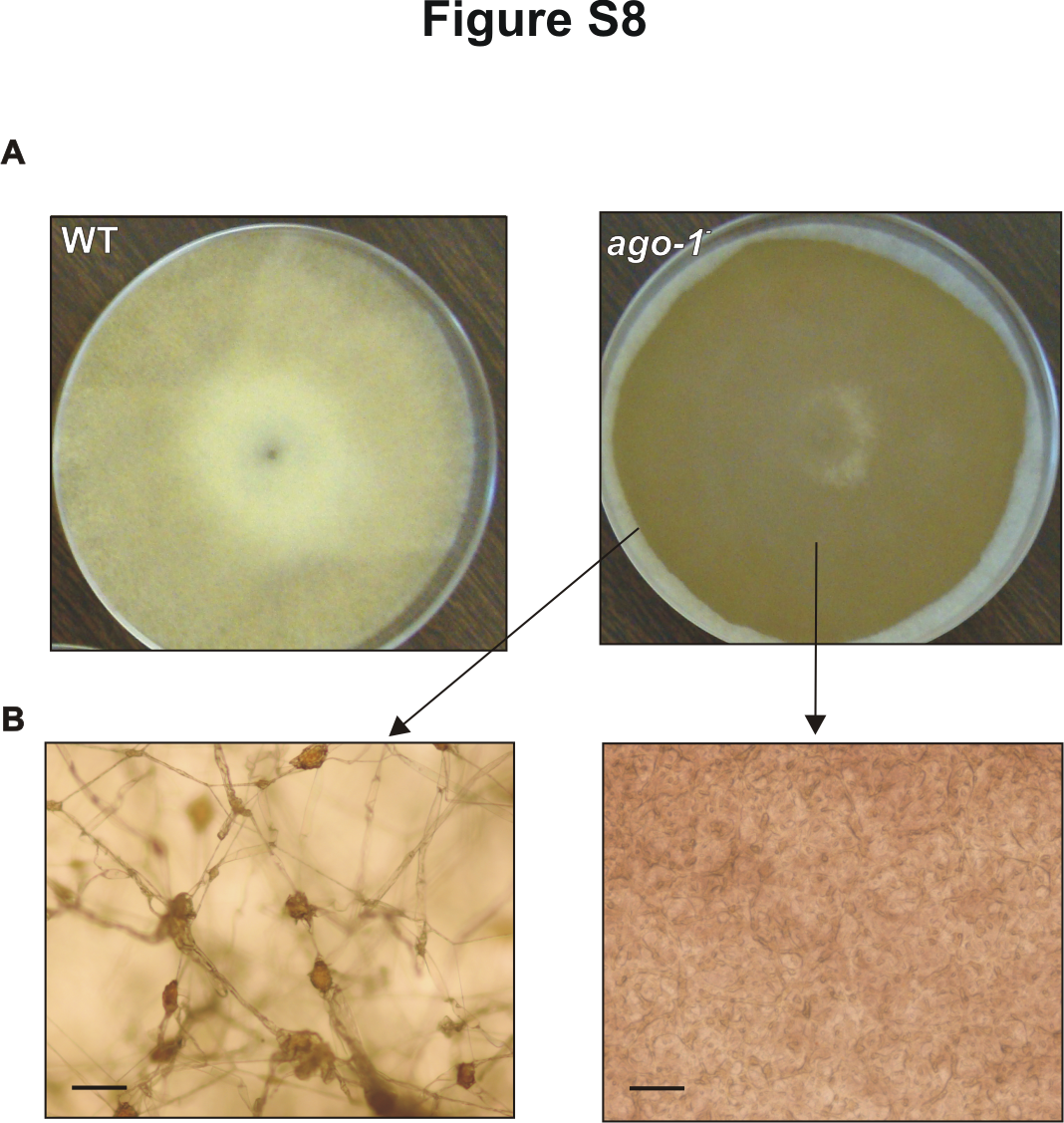

Supplement: Figure S8 — Formation of empty hyphae and hyphal degradation in aged mycelia of the ago-1 − mutant. A. Age mycelia of the ago-1 − mutant show accelerated autolysis compared to the wild type strain. B. Lysed mycelia of ago-1 − mutant present vacuolization and empty hypha in the border between the mycelial and autolysis areas (left), whereas the central part of the autolysis area is completely free of hyphal material (right). Cultures were grown for 18 days in YPD plates. Scale = 100 mm. (TIF) [file pone.0069283.s008.tif]
