# Supplementary material for: A Single Argonaute Gene Participates in Exogenous and Endogenous RNAi and Controls Cellular Functions in the Basal Fungus Mucor circinelloides
Source: PLoS One. 2013 Jul 23;8(7):e69283. doi: 10.1371/journal.pone.0069283 (PMC3720535; doi:10.1371/journal.pone.0069283)
Supplement: Table S1 — Sequences of oligonucleotides used for cloning and functional analysis of the ago genes. (PDF) [file pone.0069283.s009.pdf]

Table S1. Sequences of oligonucleotides used for cloning and functional analysis of the *ago* genes.

Gene	Name	Sequence[1]
ago-1	argo1	5'-CAACATGATCAAGGAGGTCC-3'
	argo2	5'-GTACGCTCAATGTTACCTTG-3'
	argo21	5'-GCCGGC*TCTAGA*CTGCCGACGCATAGACAACC-3'
	argo22	5'-GGCTAC*GGATCC*CACCAGCCTTCTTGATACGG-3'
	argo23	5'-GCGCCC*GGATCC*CACTTGTCAAAAGCCCAACG-3',
	argo24	5'-GCCCCG*ATCGAT*ACCCCATCACGGTAGAAAAG-3'
	argo25	5'-CTTGGGTCGTCGTTCTCTTG-3'
	argo26	5'-CCGGCC*GTCGAC*TATACACAAACAATCAAGGG-3'
	argo28	5’-GTTCGCCACGTTCCCTCTAC-3’
	argo29	5’-GGTCAGCAGGAGAGGGAGTG-3’
	argo32	5’-GCCGGCGCTAGCATGTCTTTGGAACTTACTG-3’
	argo33	5’-GAGCTTGGTGACCTTGTACTTG-3’
	argo34	5’-GAGGGGATTGGTTGGGCGTTG-3’
	argo40	5’-GCTCAC*GGGCCC*TGCCGACGCATAGACAACC-3’
ago-2	argo4	5’-TCAGCACCAGGAGCGGGATG-3’
	argo6	5’-CTCTTGCTTGATGGACATGC-3’
	argo13	5’-GGCCGTTCCACCAAGATTAG-3’
	argo27	5’-TGCTCTGTTTGCCTTCTTCG-3’
	argo38	5'-GGAGT*GGATCC*GGCGAGTTTACCATCCAAG-3'
	argo39	5'-CCAGC*GGATCC*TGCTCGTTCTGCCAAATAC-3'
	argo41	5’-GATCTAGGTGTTCGTCAAGC-3’
ago-3	argo30	5’-CTGCTATGCATGAATCTGGC-3’
	argo31	5’-TCGTCGCAAAAAGTAATCAG-3’
	argo37	5’-ACATCACAGTTTACATAGTC-3’
pyrG	pyrGZ	5’-GGCATTGGGATGCTGTTGTC-3’
	pyrG10F	5’-ATCTCAGCAGCATCGGATGG-3’

[1] Cursive nucleotides indicate restriction sites used for cloning
