# Supplementary material for: A Single Argonaute Gene Participates in Exogenous and Endogenous RNAi and Controls Cellular Functions in the Basal Fungus Mucor circinelloides
Source: PLoS One. 2013 Jul 23;8(7):e69283. doi: 10.1371/journal.pone.0069283 (PMC3720535; doi:10.1371/journal.pone.0069283)
Supplement: Table S4 — Oligonucleotides used in validation experiments. (PDF) [file pone.0069283.s012.pdf]

Table S4. Oligonucleotides used in validation experiments.

Name	Sequence	Use[1]
77050	TCTAGCCACTTGTCCTCTGGTTCA	Antisense-specific probe for esRNAs from locus 77050
78553	AGACGAGCCTATCTTCCTTGCCTTGTA	Antisense-specific probe for esRNAs from locus 78553
80452	GGTGTCTTCTGACAGTCTCGCAGAGGATGCCTCT	Antisense-specific probe for esRNAs from locus 80452
82372	TATCGCCGTCCTACCGTGGATACAA	Antisense-specific probe for esRNAs from locus 82372
86881	CGCCAATCTCCCTACAGCCTTCA	Antisense-specific probe for esRNAs from locus 86881
77442 forward	CCTCGCGAGAAAGGATTGC	Primer for DNA probe for mRNA from locus 77442
77442 reverse	GAGAACCAGATTCGGCAG	Primer for DNA probe for mRNA from locus 77442
80452 forward	CCGAAATCAGTAATGATAAGCC	Primer for DNA probe for mRNA from locus 80452
80452 reverse	GACGAAATGCTAACCGTACC	Primer for DNA probe for mRNA from locus 80452
82372 forward	GCATTGTATCCACGGTAGG	Primer for DNA probe for mRNA from locus 82372
82372 reverse	CATGCTACCCTTAGATCAGG	Primer for DNA probe for mRNA from locus 82372
83187 forward	CCACTGTCAAGCGTAAGCG	Primer for DNA probe for mRNA from locus 83187
83187 reverse	CGTATCCAGCAAGGATAGCATC	Primer for DNA probe for mRNA from locus 83187
83353 forward	GGCTACACTCGTAAGGTTCG	Primer for DNA probe for mRNA from locus 83353
83353 reverse	GGCATTGACCAGTTCACG	Primer for DNA probe for mRNA from locus 83353
95350 forward	GGACAGCATGACTGAAGGTC	Primer for DNA probe for mRNA from locus 93350
95350 reverse	CAACTCTTTGAAGCGTGGAC	Primer for DNA probe for mRNA from locus 93350

[1] Antisense-specific oligonucleotide probes for esRNAs correspond to complementary sequences to the most abundant esRNA of each locus. Primers for DNA probes for mRNA validations of the different loci amplified fragments ranging to 300-600 nt.
