# Supplementary material for: A Single Argonaute Gene Participates in Exogenous and Endogenous RNAi and Controls Cellular Functions in the Basal Fungus Mucor circinelloides
Source: PLoS One. 2013 Jul 23;8(7):e69283. doi: 10.1371/journal.pone.0069283 (PMC3720535; doi:10.1371/journal.pone.0069283)
Supplement: Material and Methods S1 — (PDF) [file pone.0069283.s015.pdf]

MATERIALS AND METHODS S1

Plasmids

Plasmid pMAT1301 contains a 5'-truncated version of the *ago-1* gene and was generated by molecular subcloning of an 8 kb *Sac*I genomic fragment isolated from a hybridizing lambda clone into the pBluescript II SK+ vector. Plasmid pMAT1333 contains the entire *ago-2* gene and was generated by molecular subcloning of a 4.3 kb *Xho*I genomic fragment isolated from a hybridizing lambda clone into the pBluescript II SK+ vector. Plasmid pMAT1305 harbours the complete *ago-3* gene and was generated by molecular subcloning of a 7 kb *Sac*I genomic fragment isolated from a hybridizing lambda clone into the pBluescript II SK+ vector. Plasmid pMAT1322 contains the complete *ago-1* gene. To construct this plasmid, genomic DNA of the *M. circinelloides* R7B strain was digested with *Aji*I and self-ligated to allow formation of circular molecules containing the known 5' *ago-1* fragment and its upstream flanking DNA. This flanking DNA was amplified using primers argo1 and argo2 (Table S1), which are complementary to regions adjacent to the upstream sequences in the circular molecules. The expected 3.1 kb fragment (estimated by Southern experiment) was cloned into the *Eco*RV site of pBluescript II SK+ vector. The resulting plasmid, pMAT1317, was then digested with *Hind*III and *Sal*I to introduce the rest of the *ago-1* gene, which was obtained by genomic PCR with primers argo25 and argo26 (Table S1), to yield plasmid pMAT1322.

Plasmid pMAT1319 was constructed to disrupt the *ago-1* gene and contains the *M. circinelloides pyrG* gene (which complements the uracil auxotrophy of the MU402 strain) flanked by *ago-1* sequences. In brief, two fragments corresponding to the 5' terminus of the *ago-1* gene and adjacent sequences (1 kb) and the 3' region of this gene (0.9 kb) were amplified from plasmids pMAT1317 and pMAT1301 using the primers pairs argo21 (*Xba*I site) and argo22 (*Bam*HI site) and argo23 (*Bam*HI site) and argo24 (*Cla*I site), respectively (Table S1). Both amplified fragments were cloned together into the pBluescript II SK+ vector

double digested with *Xba*I and *Cla*I to give plasmid pMAT1318, which contains the joined *ago-1* fragments producing a 750 bp deletion of the *ago-1* coding region. The 3.4 kb *Bam*HI fragment from pEPM1, which contains the *pyrG* gene, was cloned between the two *ago-1* fragments to produce pMAT1319. The 5.3 kb *ago-1* replacement fragment harbouring the *pyrG* gene flanked by 1 kb and 0.9 kb of *ago-1* sequences was released from pMAT1319 by *Eco*RI digestion and introduced into MU402 protoplasts by transformation.

Plasmid pMAT1338 was generated to disrupt *ago-2*. Briefly, pMAT1333 was double-digested with *Xba*I and *Cla*I, ends-repared and self-ligated to eliminate the *Bam*HI restriction site of the polylinker. The resulting plasmid was PCR amplified with primers argo38 and argo39, which included *Bam*HI sites for cloning purpose (Table S1). The PCR product contains the vector sequence flanked by 1.2 kb and 1.15 kb of *ago-2* and adjacent sequences, producing a deletion of 1.9 kb of the *ago-2* coding region. The PCR fragment was digested with *Bam*HI and ligated with the 3.4 kb *pyrG* *Bam*HI fragment to give pMAT1338. The 5.8 kb *ago-2* replacement fragment was released from pMAT1338 by *Xba*I and *Pst*I double digestion and introduced into MU402 protoplasts by transformation.

Plasmid pMAT1328 was generated to disrupt *ago-3*. The 1.6 kb *Kpn*I-*Eco*RV fragment from pMAT1305 containing the 5' terminus of *ago-3* and upstream sequences was cloned into the pBluescript II SK+ vector to give pMAT1309. The 1.2 kb *Bam*HI-*Sac*I fragment from pMAT1305 containing the 3' terminus of *ago-3* and downstream sequences was then cloned into pMAT1309 to give pMAT1310, generating a deletion of 1.37 kb of the *ago-3* coding region. The *pyrG* fragment was cloned into the *BamH*I site of pMAT1310 to give plasmid pMAT1328. The 6.2 kb *ago-3* replacement fragment was released from pMAT1328 by *Kpn*I and *Sac*I double digestion and introduced into MU402 protoplasts by transformation.

Plasmid pMAT1337 was constructed to complement the *ago-1*$^{-}$ mutation. It contains the complete *ago-1* gene and a silencing-reporter hairpin RNA construction under a strong

constitutive promoter, as well as the *M. circinelloides leuA* gene to complement the leucine auxotrophy of strain MU402. To construct this plasmid, the complete *ago-1* sequence and regulatory region was PCR amplified from pMAT1322 using universal primer T7 and primer argo40 (*Apa*I site) (Table S1). The 3.37 kb PCR product was *Apa*I digested and cloned into the *Apa*I site of pMAT1253 [9], which harbour the *carB* inverted repeat transgene as reporter of silencing and the *leu*A gene, to give plasmid pMAT1337. The *ago*-1 gene integrity was checked by sequencing.

Generation of knockout mutants for the *argonaute* genes

Knockout vectors pMAT1319, pMAT1338 and pMAT1328 were designed to disrupt *ago-1*, *ago-2* and *ago-3* genes, respectively. Restriction fragments from each plasmid containing the *pyrG* gene and sufficient sequences of the *ago* genes to allow homologous recombination were used to transform the MU402 strain (Ura^-, Leu^-). Ura^+ transformants were grown in selective medium for several vegetative cycles to increase the proportion of transformed nuclei, as primary transformants are heterokaryons due to the presence of several nuclei in the protoplasts. Homokaryotic transformants were PCR analyzed to distinguish homologous from ectopic integrations, using primer pairs pyrG10F and argo26 (*ago-1*), pyrGZ and argo41 (*ago-2*) and pyrGZ and argo37 (*ago-3*) (Table S1) to identify homologous integration events in the corresponding loci. Mutants MU413 (*ago-1*$^-$), MU416 and MU417 (*ago-2*$^-$) and MU414 and MU415 (*ago-3*$^-$) were identified. Disruption of each gene was confirmed by Southern analysis (Fig. S3). Digested DNA from each transformant was hybridized with specific *ago* probes that recognize both wild-type and *ago*$^-$ alleles but could discriminate between them (Fig. S3A-C, probes *a*, *c* and *e*). All analyzed transformants showed the expected fragments derived from the corresponding mutant alleles and the absence of the wild-type ones, confirming that those mutants have successfully replaced the wild-type *ago-1, ago-2* or *ago-3* allele (Fig.

S3D-F). The gene replacements were confirmed by hybridization with *ago* probes corresponding to the DNA region deleted in each knockout vector, which only detected the wild-type fragments (Fig. S3A-C, probes *b*, *d* and *f*). The *ago-1*$^{-}$ mutant allele has replaced 750 bp of the *ago-1* coding region by the *pyrG* gene. The *ago-2*$^{-}$ mutant allele has replaced 1.9 kb of the *ago-2* coding region by the *pyrG* gene. The *ago-3*$^{-}$ mutant allele has replaced 1.37 kb of the *ago-3* coding region by the *pyrG* gene. RT-PCR experiments confirmed lack of expression of the wild-type transcripts in the *ago-1*$^{-}$, *ago-2*$^{-}$ and *ago-3*$^{-}$ mutant strains, proving that they were all null mutants.
